# Genomic Analysis and Lineage Identification of SARS-CoV-2 Strains in Migrants Accessing Europe Through the Libyan Route

**DOI:** 10.3389/fpubh.2021.632645

**Published:** 2021-04-15

**Authors:** Fabio Tramuto, Stefano Reale, Alessandra Lo Presti, Francesco Vitale, Claudio Pulvirenti, Giovanni Rezza, Fabrizio Vitale, Giuseppa Purpari, Carmelo Massimo Maida, Salvatore Zichichi, Silvia Scibetta, Walter Mazzucco, Paola Stefanelli

**Affiliations:** ^1^Department of Health Promotion, Mother and Child Care, Internal Medicine and Medical Specialties “G. D'Alessandro”, University of Palermo, Palermo, Italy; ^2^Regional Reference Laboratory of Western Sicily for the Emergency of COVID-19, Clinical Epidemiology Unit, University Hospital “Paolo Giaccone”, Palermo, Italy; ^3^Molecular Biology Area, Zoo-prophilactic Experimental Institute of Sicily “A. Mirri”, Palermo, Italy; ^4^Department of Infectious Diseases, National Health Institute, Rome, Italy; ^5^Uffici di sanità marittima, aerea e di frontiera (USMAF) – Servizi Assistenza Sanitaria Naviganti (SASN) Sicily, Ministry of Health, Directorate-General for Health Prevention, Rome, Italy; ^6^Directorate-General for Health Prevention, Ministry of Health, Rome, Italy; ^7^Virological Diagnostic Area, Zoo-prophilactic Experimental Institute of Sicily “A. Mirri”, Palermo, Italy; ^8^Division of Biostatistics and Epidemiology, Cincinnati Children's Hospital Medical Centre, Cincinnati, OH, United States

**Keywords:** SARS-CoV-2, molecular surveillance, migrant, asylum-seeker, Mediterranean Sea, NGS

## Abstract

Many African countries, representing the origin of the majority of refugees, asylum-seekers, and other migrants, toward regions bordering on the Mediterranean area, are experiencing sustained local transmission of severe acute respiratory syndrome coronavirus 2 (SARS-CoV-2). Sicily is one of the main entry gates of migrants crossing into Europe. We conducted a pilot study, based on the full-genome sequencing of SARS-CoV-2 strains isolated from migrants coming to Sicily by crossing the Mediterranean Sea, with the aim to investigate the viral genome polymorphism and to describe their genetic variations and the phylogenetic relationships. On June 21, a nongovernmental organization vessel rescued 210 migrants crossing the Mediterranean Sea from Libya to Sicily. Of them, 13.4% tested positive for SARS-CoV-2. Eighteen whole genome sequences were obtained to explore viral genetic variability. All but one of the sequences clustered with other viral African strains within the lineage A, whereas only one intermixed among B.1 lineage genomes. Our findings documented that most of the investigated migrants acquired SARS-CoV-2 infection before landing in Sicily. However, SARS-CoV-2 transmission during travel or in overcrowded Libyan immigrant camps and/or illegal transport boats could not be ruled out. SARS-CoV-2 molecular surveillance on migrants arriving in Europe through the Sicilian gate may improve the knowledge of global SARS-CoV-2 transmission dynamic also in light of the emergence of new variants.

## Introduction

Although South Africa bears the greatest burden of disease in the continent with more than half of documented cases, other African countries, such as Nigeria, Ghana, Cameroon, Côte d'Ivoire, and Senegal, representing the origin of the majority of refugees, asylum-seekers, and other migrants toward regions bordering the Mediterranean area, are experiencing sustained local transmission of severe acute respiratory syndrome coronavirus 2 (SARS-CoV-2) (1, 2).

Sicily, being the closest territory to African borders by way of its smaller islands such as Lampedusa, is one of the first destinations of migrants crossing into Europe (3) (Supplementary Figure 1).

Between January 1, 2020 and October 21, 2020, 26,532 migrants/refugees landed in Sicily following the Libyan route by boat, either directly or after being rescued in the sea by Italian authorities or nongovernmental organizations (3), and were then hosted in dedicated reception camps or reconverted cruise ships.

Here, we report results from a pilot study based on the full-genome sequencing of SARS-CoV-2 strains isolated from migrants coming to Sicily by crossing the Mediterranean Sea in order to investigate the viral genome polymorphism, the genetic variations, and the phylogenetic relationships.

## Method

### Study Population

On June 21, a nongovernmental organization (NGO) rescue vessel saved 210 migrants near the Libyan border and arrived at the harbor of Porto Empedocle, in Southern Sicily. Of the 210 migrants, 68 (32.3%) were children or adolescents. One of the migrants, presenting with fever and respiratory symptoms, was under treatment for TB and transferred to a hospital. A rhino-pharyngeal swab, collected at hospital admission, resulted positive for SARS-CoV-2 molecular testing. Quarantine measures were implemented, and after molecular screening, 28 (13 men, nine women, and six children) out of the remaining 209 migrants resulted positive (13.4%). Of them, eight were from Cameroon, five from Guinea Conakry, three from Mali, two from Côte d'Ivoire, Sierra Leone, and Somalia, and 1 each from Nigeria, Togo, Senegal, Ghana, Liberia, and Bangladesh. The median age was 24 years. None of the migrants presented or developed signs or symptoms suggestive of COVID-19 during the follow-up.

### Ethical Approval

This study was conducted with the approval of the ethics committee of Palermo University Hospital, Palermo, Italy (n. 7/2020 released on 13/07/2020), and it is in agreement with the Helsinki Declaration.

### SARS-CoV-2 Detection and Whole Genome Sequencing

Total RNA was extracted by NucleoMag Virus (Macherey-Nagel, Germany) following the manufacturer's instructions and employing the KingFisher automatic nucleic acid extractor. SARS-CoV-2 specific targets, N1 and N2, were detected by real-time reverse transcriptase (RT)-PCR adopting primers and protocol published by the Centers for Disease Control and Prevention (CDC-006-00019, Revision: 02) (4). The probes were labeled with FAM-BHQ. PCR reactions were carried with the Brilliant III Ultra-Fast QRT-PCR Master Mix (Agilent, USA) using a QuantStudio 7 Pro Real-Time thermocycler (Thermo Fisher Scientific). Next generation sequencing (NGS) library was constructed by amplicon technique (5). Primers adopted for genome sequencing comprehended two pools, specially designed from Thermo Fisher Scientific, covering the entire genome of SARS-CoV-2. These primers are included in a package supplied by Illumina for AmpliSeq protocol (Document no. 1000000036408 v09) (5). The prepared library was purified and sequenced on MiSeq platform (Illumina). The fastq files were quality filtered and reads mapped with Bowtie2 software, against the reference genome from Wuhan (GenBank accession number NC_045512.2), to achieve the complete genome sequences. Clean genome data were visualized by IGV 2.8.0 software in order to investigate single nucleotide polymorphisms (SNPs) motives. The potentially resulting variable amino acids (AAs) in derived proteins compared to the Wuhan reference were investigated for the genomes retrieved in this study by visual inspection of the alignments.

### Phylogenetic Analysis

To explore the lineages of viruses currently circulating in the populations in the study, a selection of 18 SARS-CoV-2 genomes were obtained and analyzed, as first, through the “Pangolin COVID-19 Lineage Assigner” (6) in order to assign the lineages based on the methodology described in Rambaut (7). The assignment of the clade was also performed according to Nexstrain (8) classification. The genomes were analyzed in a phylogenetic context together with SARS-CoV-2 complete genomes from different countries, retrieved from GISAID (9) and GenBank (10), also including the above-mentioned reference sequence of the isolate Wuhan-Hu-1. Multiple nucleotide sequence alignment was performed using MAFFT v.7 (11) with the Galaxy platform (12, 13), and it was manually edited by Bioedit program (14).

The best fitting substitution model, together with the maximum likelihood (ML) phylogenetic tree, were obtained with Phyml v3.0 (14, 15). Support for the tree topology and clades was estimated with the Bayesian-like transformation of aLRT (aBayes) (16, 17). A maximum likelihood (ML) phylogenetic tree was also built with IQ-TREE software by using SH-a LRT and 1,000 number of replicates (18).

## Results

Overall, the RT-PCR assay showed SARS-CoV-2 targets with Ct values ranging from 16 to 36. All samples were included in the next massive sequencing protocol, but suitable genome libraries were recovered from 18 samples with Ct value <34. Samples showed clean mapped reads with an average coverage of the genome (referred to NC_045512.2) ranging from 158 to about 1,000. Sequencing results and coverage did not appear particularly affected by any difference of the initial Ct values.

The lineage analysis showed that the majority of the sequences from migrants (17/18, 94.4%) belonged to lineage A, while only one sequence, named EPI_ISL_582768, belonged to lineage B.1. More in depth, the clade assignment revealed that the 17 genomes belonged to clade 19B and the remaining EPI_ISL_582768 viral strain belonged to 19A clade.

**Figure 1:**
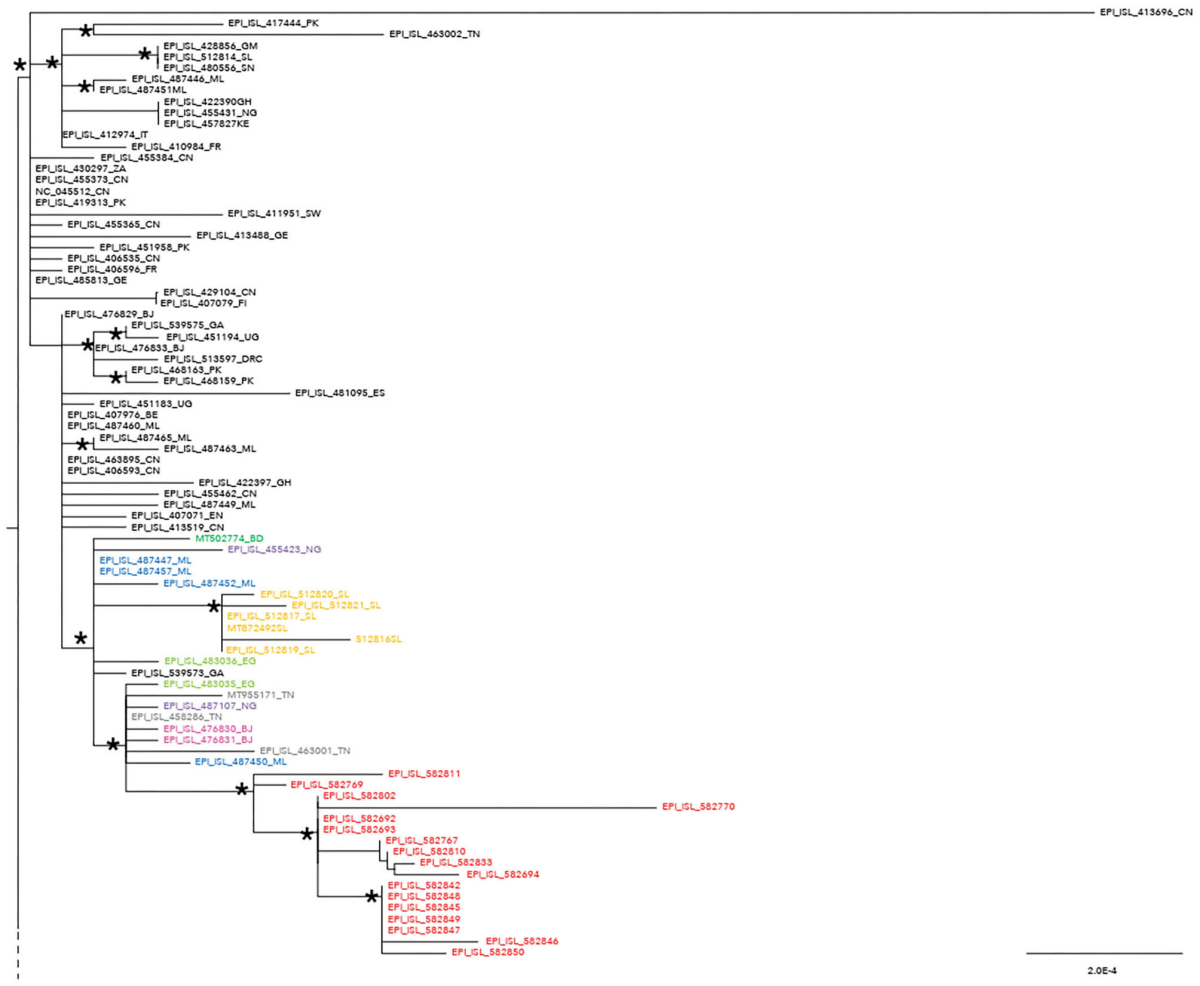
Phylogenetic analysis highlighting the selected clade extrapolated from the whole tree and focusing on the 17 severe acute respiratory syndrome coronavirus 2 (SARS-CoV-2) genomes from migrants (reported in red) belonging to lineage A. These genomes appeared related in a subclade with four genomes from Mali (ML, Mali highlighted in blue: EPI_ISl_487450; EPI_ISL_487447; EPI_ISL_487452; EPI_ISL_487457), one from Bangladesh (BD, Bangladesh reported in dark green: MT502774), two from Benin (Benin, BJ reported in fuchsia: EPI_ISL_476830 and EPI_ISL_476831), two from Nigeria (NG, Nigeria reported in light violet: EPI_ISL_487107; EPI_ISL_455423), six from Sierra Leone (SL, Sierra Leone reported in ocra yellow: EPI_ISL_512816, EPI_ISL_512817, EPI_ISL_512819, EPI_ISL_512820, EPI_ISL_512821, MT872492), three from Tunisia (TN, Tunisia reported in gray: MT955171, EPI_ISL_458286, EPI_ISL_463001), two from Egypt (EG, Egypt highlighted in light green: EPI_ISL_483035 and 483036), and one from Gabon (GA, Gabon reported in black: EPI_ISL_539573). SARS-CoV-2 genomes from other countries can be found externally located to this subclade. An ISO alpha-2 code (www.iso.org) was used at the end of the taxon names to refer to each country. An asterisk along the branches represents an aLRT–aBayes support ≥0.99 (Bayesian-like transformation of aLRT available from Phyml software) for the clade subtending that branch.

**Figure 2:**
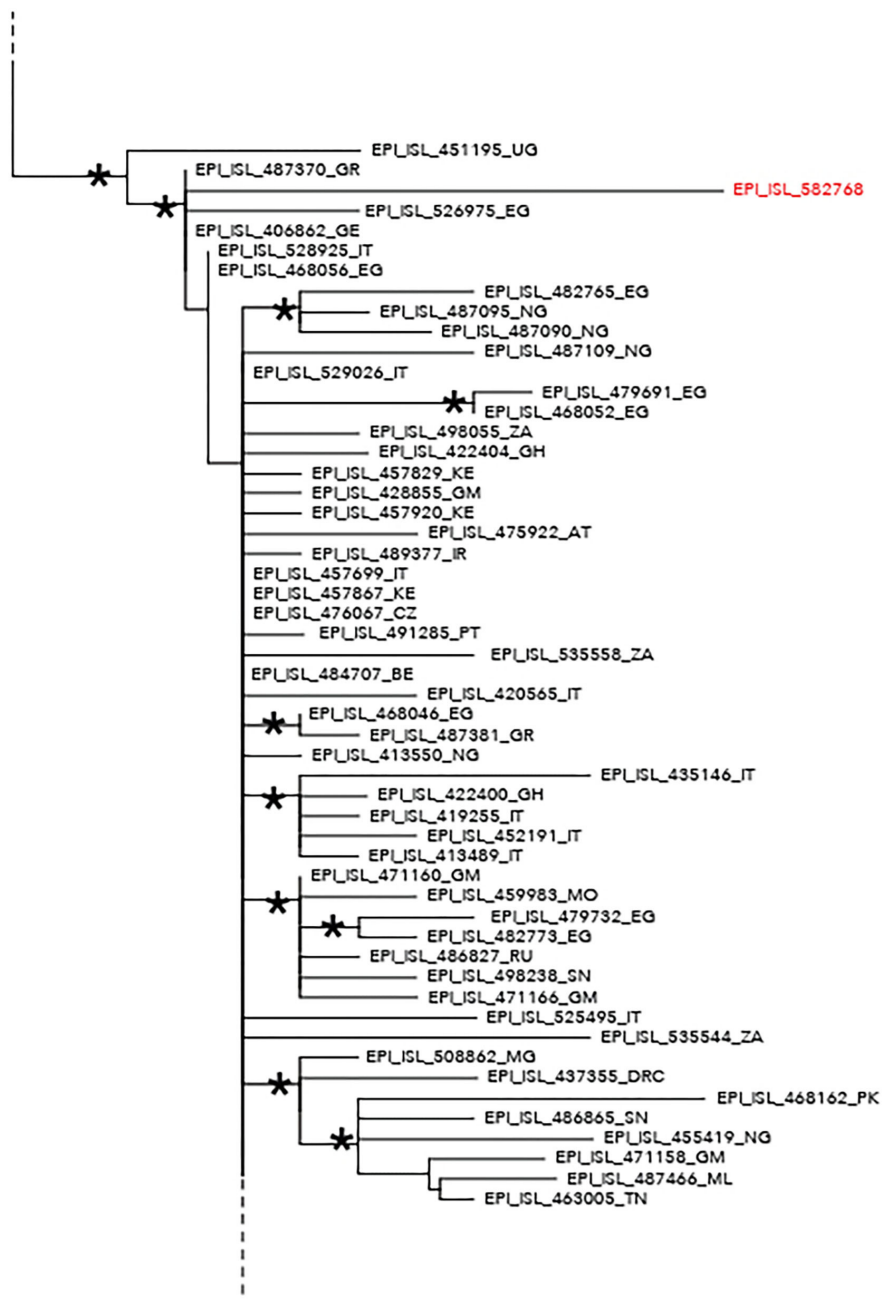
Phylogenetic analysis highlighting the selected clade extrapolated from the whole tree and including the severe acute respiratory syndrome coronavirus 2 (SARS-CoV-2) strain EPI_ISL_582768 (highlighted in red) intermixed among other SARS-CoV-2 B–B.1 lineage genomes. This strain appeared intermixed among other genomes from different countries and more proximal to strains from Egypt (EPI_ISL_526975), Greece (EPI_ISL_487370), Germany (EPI_ISL_406862), and Uganda (EPI_ISL_451195). The ISO alpha-2 codes (www.iso.org) were used at the end of the taxon names to refer to each country. An asterisk along the branches represents an aLRT–aBayes support ≥0.99 (Bayesian-like transformation of aLRT available from Phyml software) for the clade subtending that branch.

The maximum likelihood phylogenetic tree is reported as a whole in Supplementary Figure 2. Figure 1 highlights selected clades extrapolated from the whole tree including the genomes from migrants (reported in red) and belonging to lineage A (Figure 1) and lineage B.1 (Figure 2), respectively. The ML tree obtained with IQ-TREE confirmed the phylogenetic relationships above described (data not shown).

Fourteen SNPs were identified in 100% of the genomes, five of which did not involve an amino acid change and one felt into the 3′ untranslated region (UTR) region (Table 1). Amino acid mutations were located in *nsp3* (1062I, 1431V, 1612A, 1870F), in the *RNA-dependent RNA polymerase* (246I), *ORF8* (84S), and *nucleocapsid phosphoprotein* (202N, 276K) (Table 1). Two SNPs were identified in 50% of the observed sequences, generating the mutations 313F in *nsp3* and 45V in *nsp8* (Table 1).

**Table 1:** The mutation points observed in 14 of the total 18 samples (partial genomes are not included in this detection).

**Nucleotide position in whole genome sequence (Acc. Numb. NC_045512)**	***N* modif**	**Observations**	**Gene**	**Product and nt position in each respective protein sequence**	**AA in Wuhan-Hu-1 (Acc. Numb. NC_045512)**	**AA mutation identified**
361	A > **G**	100%	ORF1ab	nsp1–96 nt	32G	32G—no aa change
950	T > **C**	7%	ORF1ab	nsp 2–nt 145	49Y	49H
1427	C > **T**	7%	ORF1ab	nsp 2–nt 622	208H	208Y
3656	C > **T**	50%	ORF1ab	nsp 3–nt 937	313L	313F
5903	G > **A**	100%	ORF1ab	nsp3–nt 3184	1062V	1062I
7011	C > **T**	100%	ORF1ab	nsp3–nt 4292	1431A	1431V
7554	T > **C**	100%	ORF1ab	nsp3–nt 4835	1612V	1612A
8327	C > **T**	100%	ORF1ab	nsp 3–nt 5608	1870L	1870F
8782	C > **T**	100%	ORF1ab	nsp4–nt 228	76S	76S—no aa change
8881	G > **A**	7%	ORF1ab	nsp 4–nt 327	109T	109T—no aa change
12225	C > **T**	50%	ORF1ab	nsp8–nt 134	45A	45V
14053	C > **T**	7%	ORF1ab	RNA-dependent RNA polymerase–nt 613	205L	205L—no aa change
14064	T > **C**	7%	ORF1ab	RNA-dependent RNA polymerase–nt 624	208D	208D—no aa change
14177	C > **T**	100%	ORF1ab	RNA-dependent RNA polymerase–nt 737	246T	246I
22468	G > **T**	100%	S	Spike–nt 906	302T	302T—no aa change
23695	T > **A**	100%	S	Spike–nt 2133	711S	711S—no aa change
24588	C > **T**	7%	S	Spike–nt 3026	1009T	1009I
28144	T > **C**	100%	ORF 8	ORF 8 protein–nt 251	84L	84S
28870	A > **G**	7%	N	Nucleocapsid phosphoprotein–nt 597	199P	199P—no aa change
28878	G > **A**	100%	N	Nucleocapsid phosphoprotein–nt 605	202S	202N
29100	G > **A**	100%	N	Nucleocapsid phosphoprotein–nt 827	276R	276K
29449	G > **T**	100%	N	Nucleocapsid phosphoprotein–nt 1176	392V	392V—no aa change
29742	G > **A**	100%	3′ UTR–stem loop	/	/	/

*Bold values means modified nucleotides*.

Seven SNPs were identified in only one genome (7%, *n* = 1/14), and four of these have not determined AA changes. Among those involving AA variations, we found the substitutions 49H and 208Y in *nsp2* and 1009I in the *spike* (Table 1).

We also analyzed which of the SNPs identified in the genomes from migrants were also present in the genomes from the African countries highlighted by colors (Figure 1) and located in the same supported subclade. We therefore found three specific SNPs confirmed also in the genomes from the African countries. In particular, SNPs at the nucleotide (nt) position 361 (38%, *n* = 8/21), 8782 (95%, *n* = 20/21), and 22,468 (95%, *n* = 20/21).

The EPI_ISL_582768 revealed five SNPs that did not cause AA changes: one was located inside the 5′ UTR; meanwhile, four SNPs determined AA change (Table 2). Among those involving AA change, the first determined the mutation 21M in *nsp3*, the second the mutation 216F in *nsp3*, the third the mutation 277S in *nsp6*, and the fourth the mutation 614G in the *spike* (Table 2).

**Table 2:** The mutation points observed in the sample EPI_ISL_582768.

**Nucleotide position in whole genome sequence (Acc. Numb. NC_045512)**	***N* modif**	**Gene**	**Product and nt position in each respective protein sequence**	**AA in Wuhan-Hu-1 (Acc. Numb. NC_045512)**	**AA mutation identified**
241	C > **T**	5′ UTR	/	/	/
475	C > **T**	ORF1ab	nsp1–nt 210	70F	70F—no aa change
2780	G > **A**	ORF1ab	nsp 3- nt 61	21V	21M
3037	C > **T**	ORF1ab	nsp3–nt 318	106F	106F—no aa change
3365	C > **T**	ORF1ab	nsp 3–nt 646	216L	216F
7210	A > **G**	ORF1ab	nsp3–nt 4491	1497K	1497K—no aa change
11801	G > **A**	ORF1ab	nsp6–nt 829	277G	277S
22882	T > **C**	S	Spike–nt 1320	440N	440N—no aa change
23403	A > **G**	S	Spike–nt 1841	614D	614G
26936	C > **T**	M	Membrane glycoprotein–nt 414	138L	138L—no aa change

*Bold values means modified nucleotides*.

## Discussion

The first case of COVID-19 was reported in the African continent on February 14, 2020 (19). Nevertheless, because of low-to-absent testing capacity and poor reporting systems, to date, limited information are available on the burden of COVID-19 and the genetic characteristics of SARS-CoV-2 viruses circulating in Africa (2, 20).

We investigated the viral genome polymorphism of SARS-CoV-2 genomes isolated from a sample of migrants coming to Sicily by crossing the Mediterranean Sea, following the Libyan route, and hosted in dedicated reception centers (21). Our analysis identified some genomic lineages previously detected in different low-income countries. In particular, the majority of the genomes here investigated from migrants belonged to lineage A (only one sequence belonged to lineage B.1).

Despite the several limitations related to the convenient sample and to the lack of available genomes from each African country, phylogenetic relationships and SNPs analyses were carried out.

Phylogenetic analysis consistently placed the migrant genomes, except for one, in a supported subclade grouping with viral African genomes (lineage A) identified in Mali, Bangladesh, Benin, Nigeria, Sierra Leone, Tunisia, Egypt, and Gabon. The EPI_ISL_582768 clustered in a different clade, intermixed among B-B.1 lineage genomes from various countries, and more proximal to strains from Egypt, Greece, Uganda, and Germany.

The unique sample clustering among B–B.1 lineage genomes exhibited a signature mutation profile near to ST4 [previously described in Yang et al. (22)] that includes three SNPs: C241T, C3037T, and A23403G. In Africa, ST4 has been reported for cases reporting travel history to Europe (23). Moreover, lineage B.1 was described in some African countries, due to returning travelers (20). As reported, several hypotheses could be in support to the origin of the infection of the only one genome belonging to the B.1 lineage.

In agreement with previous data (8, 9, 24, 25), we highlighted in the lineage A isolates from migrants the two very stable SNPs, i.e., C8782T and T28144C, previously reported to be marker variant and specific of clade S–lineage A (7). This finding is consistent with the highest frequencies of lineage A previously reported in Africa (93%) (25).

The genetic variability due to the presence of SNPs associated with the different important encoding proteins, have been, at least in part, previously reported (8, 9, 26–28). Most of them have to be carefully monitored as a crucial role in the evolution of SARS-CoV-2. Specifically, mutations in the spike gene and in the RNA-dependent RNA polymerase may have a role as target for vaccine design and antiviral treatment.

Overall, we hypothesize that migrants have acquired SARS-CoV-2 infection before landing in Sicily. However, SARS-CoV-2 transmission during travel or in overcrowded Libyan immigrant camps and/or illegal transport boats could not be ruled out (29).

These findings support the use of extensive genomic surveillance of SARS-CoV-2 among asylum-seekers arriving in Italy through the Sicilian gate also in light of the emergence of new variants (30). Migrant reception camps may provide an opportunity to improve knowledge on SARS-CoV-2 dynamic in “neglected” geographical areas and on genetic diversity and phylogenetic relationships in order to improve prevention and control programs for vulnerable populations (31, 32).

Lastly, the study of virus genetic variations in poorly resourced countries and their evolutionary trajectories may be useful for global SARS-CoV-2 transmission dynamics (20).

## Data Availability Statement

The original contributions presented in the study are publicly available. This data can be found here: The genome sequences were deposited into GenBank database with accession numbers from MW340787 to MW340802.

## Ethics Statement

The studies involving human participants were reviewed and approved by Ethics committee of Palermo University Hospital, Palermo, Italy. Written informed consent from the participants' legal guardian/next of kin was not required to participate in this study in accordance with the national legislation and the institutional requirements.

## Author Contributions

FT, FrV, FaV, WM, and PS: methodology. SR, SS, and AL: formal analysis. All authors: investigation and writing, review, and editing.

## Conflict of Interest

The authors declare that the research was conducted in the absence of any commercial or financial relationships that could be construed as a potential conflict of interest.

## Supplementary Material

Supplementary Figure 1 Main route followed by migrants to Europe through the Sicilian gate.

Supplementary Figure 2 Maximum likelihood phylogenetic analysis of 18 SARS-CoV-2 genomes from migrants (reported in red) and 262 SARS-CoV-2 complete genomes from different countries, retrieved from GISAID and GenBank obtained with the best fitting substitution model with Phyml v3.0. The tree was midpoint rooted. The scale bar at the bottom of the tree represents 0.0002 nucleotide substitution per site. The ISO alpha−2 codes (www.iso.org) were used at the end of the taxon names to refer to each country. An asterisk along the branches represents an aLRT - aBayes support ≥0.99 (Bayesian-like transformation of aLRT available from Phyml software) for the clade subtending that branch. The African genomes located in the sub-clade in the upper part of the tree were highlighted in different colors (as described in Figure 1).

Supplementary Table 1 Sequences used in this study and acknowledgment table.
