# Supplementary figures and images for: Genomic Analysis and Lineage Identification of SARS-CoV-2 Strains in Migrants Accessing Europe Through the Libyan Route
Source: Front Public Health. 2021 Apr 15;9:632645. doi: 10.3389/fpubh.2021.632645 (PMC8082074; doi:10.3389/fpubh.2021.632645)

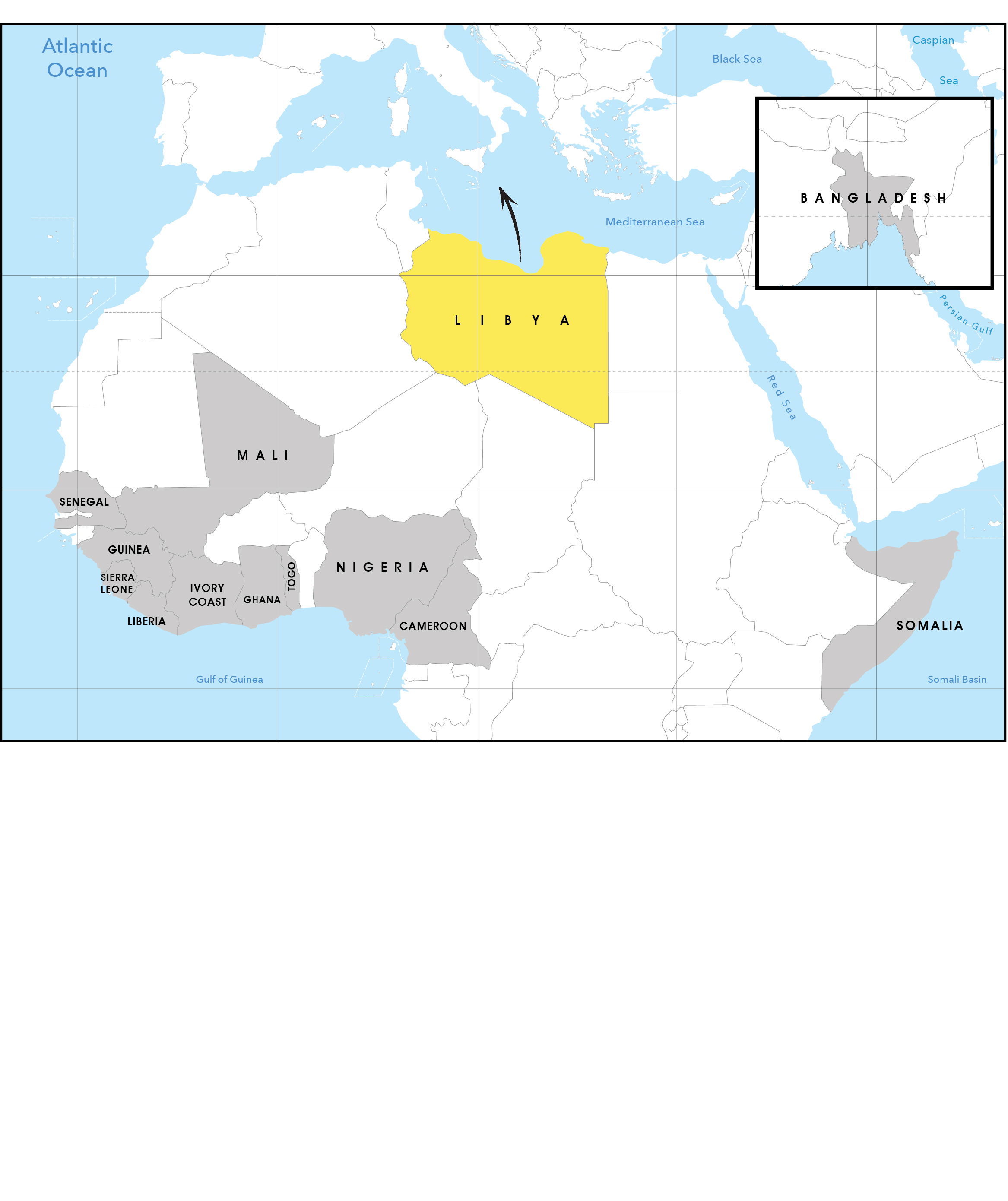

Supplement: Supplementary Figure 1 — Main route followed by migrants to Europe through the Sicilian gate. [file Image_1.JPEG]

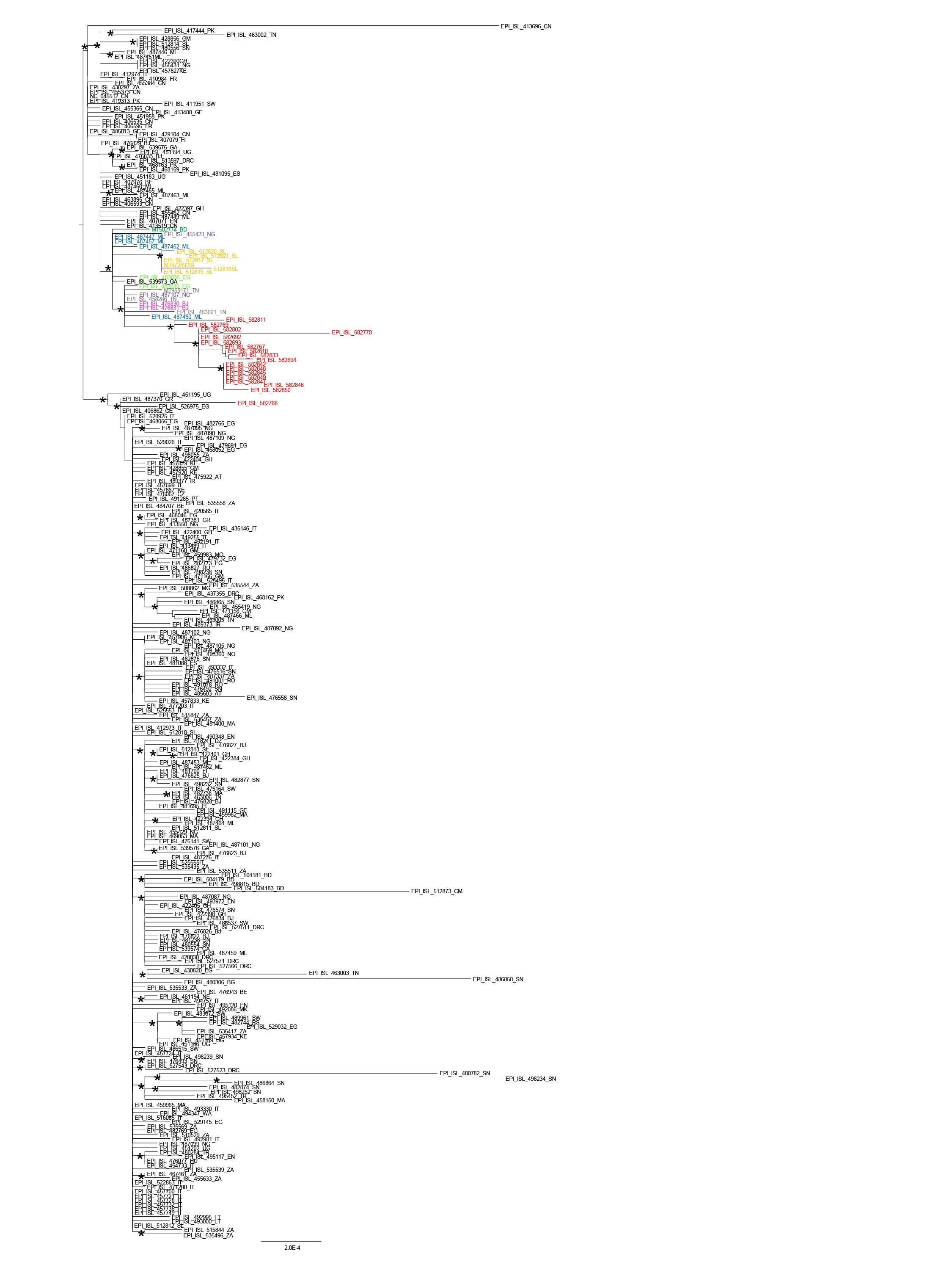

Supplement: Supplementary Figure 2 — Maximum likelihood phylogenetic analysis of 18 SARS-CoV-2 genomes from migrants (reported in red) and 262 SARS-CoV-2 complete genomes from different countries, retrieved from GISAID and GenBank obtained with the best fitting substitution model with Phyml v3.0. The tree was midpoint rooted. The scale bar at the bottom of the tree represents 0.0002 nucleotide substitution per site. The ISO alpha−2 codes (www.iso.org) were used at the end of the taxon names to refer to each country. An asterisk along the branches represents an aLRT - aBayes support ≥0.99 (Bayesian-like transformation of aLRT available from Phyml software) for the clade subtending that branch. The African genomes located in the sub-clade in the upper part of the tree were highlighted in different colors (as described in Figure 1). [file Image_2.JPEG]
