# Supplementary material for: Genomic Analysis and Lineage Identification of SARS-CoV-2 Strains in Migrants Accessing Europe Through the Libyan Route
Source: Front Public Health. 2021 Apr 15;9:632645. doi: 10.3389/fpubh.2021.632645 (PMC8082074; doi:10.3389/fpubh.2021.632645)
Supplement: Supplementary Table 1 — Sequences used in this study and acknowledgment table. [file Table_1.DOCX]

***Supplementary Table S1. Sequences used in this study and Acknowledgment Table.***

*We gratefully acknowledge the Authors, the Originating and Submitting Laboratories for their sequence and metadata shared through GISAID, on which this research is based. All submitters of data may be contacted directly via* [*www.gisaid.org*](http://www.gisaid.org)*. The table shows Accession ID, Originating Lab, Submitting Lab, Authors*

- **Accession ID:** EPI_ISL_406535 - **Originating Laboratory:** Guangdong Provincial Center for Diseases Control and Prevention; Guangdong Provincial Public Health – **Submiting Laboratory:** Guangdong Provincial Center for Diseases Control and Prevention – **Authors:** Min Kang, Jie Wu, Jing Lu, Tao Liu, Baisheng Li, Shujiang Mei, Feng Ruan, Lifeng Lin, Changwen Ke, Haojie Zhong, Yingtao Zhang, Lirong Zou, Xuguang Chen, Qi Zhu, Jianpeng Xiao, Jianxiang Geng, Zhe Liu, Jianxiong Hu, Weilin Zeng, Xing Li, Yuhuang Liao, Xiujuan Tang, Songjian Xiao, Ying Wang, Yingchao Song, Xue Zhuang, Lijun Liang, Guanhao He, Huihong Deng, Tie Song, Jianfeng He, Wenjun Ma.
- **Accession ID:** EPI_ISL_406593 - **Originating Laboratory:** Shenzhen Key Laboratory of Pathogen and Immunity, National Clinical Research Center for Infectious Disease, Shenzhen Third People's Hospital– **Submiting Laboratory:** Shenzhen Key Laboratory of Pathogen and Immunity, National Clinical Research Center for Infectious Disease, Shenzhen Third People's Hospital – **Authors:** Yang Yang, Chenguang Shen, Li Xing, Zhixiang Xu, Haixia Zheng, Yingxia Liu
- **Accession ID:** EPI_ISL_406596 - **Originating Laboratory:** Department of Infectious and Tropical Diseases, Bichat Claude Bernard Hospital, Paris – **Submiting Laboratory:** National Reference Center for Viruses of Respiratory Infections, Institut Pasteur, Paris – **Authors:** Mélanie Albert, Marion Barbet, Sylvie Behillil, Méline Bizard, Angela Brisebarre, Flora Donati, Vincent Enouf, Maud Vanpeene, Sylvie van der Werf, Yazdan Yazdanpanah, Xavier Lescure.
- **Accession ID:** EPI_ISL_406862 - **Originating Laboratory:** Charité Universitätsmedizin Berlin, Institute of Virology; Institut für Mikrobiologie der Bundeswehr, Munich – **Submiting Laboratory:** Charité Universitätsmedizin Berlin, Institute of Virology – **Authors:** Victor M Corman, Julia Schneider, Talitha Veith, Barbara Mühlemann, Markus Antwerpen, Christian Drosten, Roman Wölfel.
- **Accession ID:** EPI_ISL_407071 - **Originating Laboratory:** Respiratory Virus Unit, Microbiology Services Colindale, Public Health England – **Submiting Laboratory:** Respiratory Virus Unit, Microbiology Services Colindale, Public Health England - **Authors:** Monica Galiano, Shahjahan Miah, Richard Myers, Angie Lackenby, Omolola Akinbami, Tiina Talts, Leena Bhaw, Kirstin Edwards, Jonathan Hubb, Joanna Ellis, Maria Zambon
- **Accession ID:** EPI_ISL_407079 - **Originating Laboratory:** Lapland Central Hospital – **Submiting Laboratory:** Department of Virology, University of Helsinki and Helsinki University Hospital, Helsinki, Finland - **Authors:** Teemu Smura, Suvi Kuivanen, Hannimari Kallio-Kokko, Olli Vapalahti
- **Accession ID:** EPI_ISL_407976 - **Originating Laboratory:** KU Leuven, Clinical and Epidemiological Virology – **Submiting Laboratory:** KU Leuven, Clinical and Epidemiological Virology - **Authors:** Bert Vanmechelen, Elke Wollants, Annabel Rector, Els Keyaerts, Lies Laenen, Marc Van Ranst, and Piet Maes
- **Accession ID:** EPI_ISL_420565 **- Originating Laboratory:** Ospedale Civile Giuseppe Mazzini**– Submiting Laboratory:** Istituto Zooprofilattico Sperimentale dell'Abruzzo e Molise "G. Caporale" Teramo – Italy - **Authors:** Lorusso A, Marcacci M, Di Domenico M, Ancora M, Curini V, Mangone I, Rinaldi A, Di Pasquale A, Cammà C, Puglia I, Savini G
- **Accession ID:** EPI_ISL_410984 - **Originating Laboratory:** Department of Infectious and Tropical Diseases, Bichat Claude Bernard Hospital, Paris – **Submiting Laboratory:** National Reference Center for Viruses of Respiratory Infections, Institut Pasteur, Paris - **Authors:** Mélanie Albert, Marion Barbet, Sylvie Behillil, Méline Bizard, Angela Brisebarre, Flora Donati, Vincent Enouf, Maud Vanpeene, Sylvie van der Werf, Yazdan Yazdanpanah, Xavier Lescure
- **Accession ID:** EPI_ISL_411951 - **Originating Laboratory:** Unit for Laboratory Development and Technology Transfer, Public Health Agency of Sweden – **Submiting Laboratory:** Unit for Laboratory Development and Technology Transfer, Public Health Agency of Sweden - **Authors:** Bengner,M., Palmerus,M., Lindsjo,O., Lind Karlberg,M., Monteil,V., Appelberg,S., Brave,A., Muradrasoli,S. and Tegmark-Wisell,K.
- **Accession ID:** EPI_ISL_413488 - **Originating Laboratory:** Center of Medical Microbiology, Virology, and Hospital Hygiene, University of Duesseldorf – **Submiting Laboratory:** Center of Medical Microbiology, Virology, and Hospital Hygiene, University of Duesseldorf - **Authors:** Ortwin Adams, Marcel Andree, Alexander Dilthey, Torsten Feldt, Sandra Hauka, Torsten Houwaart, Björn-Erik Jensen, Detlef Kindgen-Milles, Malte Kohns Vasconcelos, Klaus Pfeffer, Tina Senff, Daniel Strelow, Jörg Timm, Andreas Walker, Tobias Wienemann
- **Accession ID:** EPI_ISL_413489 - **Originating Laboratory:** Laboratorio di Microbiologia e Virologia, Università Vita-Salute San Raffaele, Milano – **Submiting Laboratory**: Laboratorio di Microbiologia e Virologia, Università Vita-Salute San Raffaele, Milano - **Authors:** R.A Diotti, E. Criscuolo, M. Castelli, V. Caputo, R. Ferrarese, M. Sampaolo, E. Boeri, I. Negri, V. Amato, G. Lo Raso, C. Di Resta, R. Burioni, M. Clementi, N. Mancini & N. Clementi
- **Accession ID:** EPI_ISL_413519 - **Originating Laboratory:** Infectious Disease Control Center, Center for Disease Control and Prevention of PLA – **Submiting Laboratory:** Infectious Disease Control Center, Center for Disease Control and Prevention of PLA **- Authors:** Li,J., Li,L., Li,Z., Qiu,S., Song,H., Li,P. and Li,P.
- **Accession ID:** EPI_ISL_413696 - **Originating Laboratory:** Weifang Center for Disease Control and Prevention – **Submiting Laboratory:** Weifang Center for Disease Control and Prevention & BGI-Shenzhen - **Authors:** Qing Nie, Xingguang Li, Erik M Volz, Han Fu, Haowei Wang, Xiaoyue Xi, Wei Chen, Dehui Liu, Yingying Chen, Mengmeng Tian, Wei Tan, Junjie Zai, Wanying Sun, Jiandong Li, Junhua Li
- **Accession ID:** EPI_ISL_418241- **Originating Laboratory:** NIC Viral Respiratory Unit - Institut Pasteur of Algeria – **Submiting Laboratory:** National Reference Center for Viruses of Respiratory Infections, Institut Pasteur, Paris - **Authors:** Mélanie Albert, Marion Barbet, Sylvie Behillil, Méline Bizard, Angela Brisebarre, Flora Donati, Etienne Simon-Lorière, Vincent Enouf, Maud Vanpeene, Sylvie van der Werf, Fawzi Derrar
- **Accession ID:** EPI_ISL_419255 - **Originating Laboratory:** INMI Lazzaro Spallanzani IRCCS – **Submiting Laboratory:** INMI Lazzaro Spallanzani IRCCS **- Authors:** Antonino Di Caro, Cesare E. M. Gruber, Martina Rueca, Barbara Bartolini, Francesco Messina, Emanuela Giombini, Maria R. Capobianchi, Fabrizio Carletti, Francesca Colavita, Concetta Castilletti, Eleonora Lalle, Daniele Lapa, Giuseppe Ippolito.
- **Accession ID:** EPI_ISL_420030 - **Originating Laboratory:** Viral Respiratory Lab, National Institute for Biomedical Research (INRB) – **Submiting Laboratory:** Pathogen Sequencing Lab, National Institute for Biomedical Research (INRB) **- Authors:** Placide Mbala-Kingebeni, Edith Nkwembe, Eddy Kinganda-Lusamaki, Amuri Aziza, Catherine Pratt, Matthias Pauthner, Josh Quick, Allison Black, James Hadfield, Trevor Bedford, Ian Goodfellow, Nick Loman, Kristian Andersen, Michael Wiley, Steve Ahuka-Mundeke, Jean-Jacques Muyembe Tamfum.
- **Accession ID:** EPI_ISL_429104 - **Originating Laboratory:** The First Affiliated Hospital of Guangzhou Medical University – **Submiting Laboratory:** BGI-shenzhen & The First Affiliated Hospital of Guangzhou Medical University - **Authors**: - **Submitter**: Ji, Jingkai
- **Accession ID:** EPI_ISL_430820 - **Originating Laboratory:** Center of Scientific Excellence for Influenza Viruses, National Research Centre (NRC), Egypt. – **Submiting Laboratory:** Center of Scientific Excellence for Influenza Viruses, National Research Centre (NRC), Egypt. - **Authors:** Mohamed Ahmed Ali, Ahmed Kandeil, Ahmed Mostafa, Rabeh El-Shesheny, Mahmoud Shehata, Wael Roshdy, Shymaa Showky Ahmed , Amal Naguib, Mokhtar Gomaa, Ahmed El-Taweel, Ahmed E Kayed, Yassmin Moatasim, Omnia Kutkat, Sara Mahmoud, Mina Kamel, Abo Shama, M Noura, Mohamed El Sayes, Nancy M. El Guindy.
- **Accession ID:** EPI_ISL_435146 - **Originating Laboratory:** Villa Serena del Dr. Leonardo Petruzzi – **Submiting Laboratory:** Istituto Zooprofilattico Sperimentale dell'Abruzzo e Molise "G. Caporale" **- Authors:** Lorusso A, Marcacci M, Di Domenico M, Ancora M, Curini V, Mangone I, Rinaldi A, Di Pasquale A, Cammà C, Puglia I, Savini G.
- **Accession ID:** EPI_ISL_437355 - **Originating Laboratory:** Viral Respiratory Lab, National Institute for Biomedical Research (INRB) – **Submiting Laboratory:** Pathogen Sequencing Lab, National Institute for Biomedical Research (INRB) **- Authors:** Placide Mbala-Kingebeni, Edith Nkwembe, Eddy Kinganda-Lusamaki, Amuri Aziza, Francisca Muyembe Mawete, Catherine Pratt, Matthias Pauthner, Josh Quick, Allison Black, James Hadfield, Trevor Bedford, Ian Goodfellow, Andrew Rambaut, Nick Loman, Kristian Andersen, Michael Wiley, Steve Ahuka-Mundeke, Jean-Jacques Muyembe Tamfum.
- **Accession ID:** EPI_ISL_452191 - **Originating Laboratory:** ULSS9 Distretto di San Bonifacio – **Submiting Laboratory:** Istituto Zooprofilattico Sperimentale delle Venezie **- Authors:** Adelaide Milani, Alessia Schivo, Annalisa Salviato, Erika Giorgia Quaranta, Gianpiero Zamperin, Ambra Pastori, Bianca Zecchin, Alice Fusaro, Calogero Terregino, Antonia Ricci.
- **Accession ID:** EPI_ISL_454733 - **Originating Laboratory:** Department of Medical, Biotechnologies University of Siena – **Submiting Laboratory:** Department of Medical, Biotechnologies University of Siena - **Authors:** Cusi, M.G., Pinzauti, D., Gandolfo,C., Anichini,G., Pozzi,G. and Santoro,F.
- **Accession ID:** EPI_ISL_455365 - **Originating Laboratory:** Wuhan Chain Medical Labs (CMLabs) – **Submiting Laboratory:** State Key Laboratory of Biotherapy of Sichuan University **- Authors**: Baowen Du, Minjin Wang, Chao Tang, Chuan Chen, Yongzhao Zhou, Mingxia Yu, Hancheng Wei, Weimin Li, Jing-wen Lin, Jia Geng, Binwu Ying, Lu Chen.
- **Accession ID:** EPI_ISL_455373 - **Originating Laboratory:** Wuhan Chain Medical Labs (CMLabs) – **Submiting Laboratory:** State Key Laboratory of Biotherapy of Sichuan University **- Authors:** Baowen Du, Minjin Wang, Chao Tang, Chuan Chen, Yongzhao Zhou, Mingxia Yu, Hancheng Wei, Weimin Li, Jing-wen Lin, Jia Geng, Binwu Ying, Lu Chen.
- **Accession ID:** EPI_ISL_455384 - **Originating Laboratory:** Wuhan Chain Medical Labs (CMLabs) – **Submiting Laboratory:** State Key Laboratory of Biotherapy of Sichuan University **- Authors:** Baowen Du, Minjin Wang, Chao Tang, Chuan Chen, Yongzhao Zhou, Mingxia Yu, Hancheng Wei, Weimin Li, Jing-wen Lin, Jia Geng, Binwu Ying, Lu Chen.
- **Accession ID:** EPI_ISL_455462 - **Originating Laboratory:** Jiangxi Province Center for Disease Control and Prevention – **Submiting Laboratory:** Jiangxi Province Center for Disease Control and Prevention - **Authors:** JianXiong Li,Ying Xiong,Tian Gong,Yong Shi,Jun Zhou,Fang Xiao,ShiWen Liu,XiaoQing Liu,Gang Xu,DaJin Xiao,Xin Ran,YanNi Zhang.
- **Accession ID:** EPI_ISL_457721 - **Originating Laboratory:** Department of Infectious Diseases, Istituto Superiore di Sanità, Roma, Italy – **Submiting Laboratory:** Army Medical and Veterinary Research Center **- Authors:** Paola Stefanelli, Alessandra Lo Presti, Stefano Fiore, Antonella Marchi, Eleonora Benedetti, Concetta Fabiani Silvia Fillo, Giovanni Faggioni, Riccardo De Sanctis, Antonella Fortunato, Anna Anselmo, Francesco Giordani, Vanessa Vera Fain, Nino D’Amore, Florigio Lista.
- **Accession ID:** EPI_ISL_461194 - **Originating Laboratory:** Dutch COVID-19 response team – **Submiting Laboratory:** Erasmus Medical Center **- Authors:** Bas Oude Munnink, David Nieuwenhuijse, Reina Sikkema, Claudia Schapendonk, Irina Chestakova, Anne van der Linden, Theo Bestebroer, Stefan van Nieuwkoop, Mark Pronk, Pascal Lexmond, Corien Swaan, Manon Haverkate, Madelief Mollers, Mart Stein, Sandra Kengne Kamga Mobou, Jeroen van Kampen, Jolanda Voermans, Aura Timen, Corine GeurtsvanKessel, Annemiek van der Eijk, Richard Molenkamp, Marion Koopmans, on behalf of the Dutch national COVID-19 response team.
- **Accession ID:** EPI_ISL_463895 - **Originating Laboratory:** Shaoxing Center for Disease Control and Prevention – **Submiting Laboratory:** Department of Pathology and Laboratory Medicine, University of California Los Angeles - **Authors:** Jinkun Chen, Evann E. Hilt, Huan Wu, Zhuojing Jiang, QinChao Zhang, JiLing Wang, Yifang Wang, Fan Li, Ziqin Li, Jialiang Tang, Shangxin Yang.
- **Accession ID:** EPI_ISL_468046 - **Originating Laboratory:** Egyptian National Cancer Institute (ENCI) – **Submiting Laboratory:** Egyptian National Cancer Institute (ENCI) **- Authors:** Zekri, Abdel Rahman N, Amer,K.E., Ahmed,O.S., Soliman,H.K., Hafez,M.M., Bahnassy,A.A., Abdelhamid,W., Gad,A., Ali,M., Hassan,W., Samir,M., Raouf,A., Hamdy,M.S., Soliman,M.S., Elsissy,M.H., Elkhateeb,S.M., Ezzelarab,M.H., Abouelhoda, Mohamed.
- **Accession ID:** EPI_ISL_468052 - **Originating Laboratory:** Egyptian National Cancer Institute (ENCI) – **Submiting Laboratory:** Egyptian National Cancer Institute (ENCI) **- Authors:** Zekri, Abdel Rahman N, Amer,K.E., Ahmed,O.S., Soliman,H.K., Hafez,M.M., Bahnassy,A.A., Abdelhamid,W., Gad,A., Ali,M., Hassan,W., Samir,M., Raouf,A., Hamdy,M.S., Soliman, M.S., Elsissy, M.H., Elkhateeb, S.M., Ezzelarab, M.H., Abouelhoda, Mohamed.
- **Accession ID:** EPI_ISL_468056 - **Originating Laboratory:** Egyptian National Cancer Institute (ENCI) – **Submiting Laboratory:** Egyptian National Cancer Institute (ENCI) **- Authors:** Zekri, Abdel Rahman N., Amer,K.E., Ahmed,O.S., Soliman,H.K., Ali,M.A., Hassan,W.A., Mahmoud,A.A., Khattab,A.A., Hafez,M.M., Abouelhoda, Mohamed.
- **Accession ID:** EPI_ISL_475164 - **Originating Laboratory:** Halmstad klinisk mikrobiologi – **Submiting Laboratory:** The Public Health Agency of Sweden **- Authors:** Oskar Karlsson Lindsjo, Maria Lind Karlberg, Mattias Haukland, Reza Advani, Olov Svartstrom, Anna-Malin Linde, Sandra Broddesson, Petra Edquist, Shamam Muradrasoli, Anna Risberg, Karin Tegmark-Wisell.
- **Accession ID:** EPI_ISL_475922 - **Originating Laboratory**: Institut für Virologie am Department für Hygiene, Mikrobiologie und Public Health – **Submiting Laboratory:** Bergthaler laboratory, CeMM Research Center for Molecular Medicine of the Austrian Academy of Sciences **- Authors:** Alexandra Popa, Benedikt Agerer, Henrique Colaco, Lukas Endler, Jakob-Wendelin Genger, Alexander Lercher, Mark Smyth, Thomas Penz, Michael Schuster, Jan Laine, Martin Senekowitsch, Judith Aberle, Stephan Aberle, Peter Hufnagl, Daniela Schmid, Franz Allerberger, Elisabeth Puchhammer-Stoeckl, Manfred Nairz, Guenter Weiss, Gregor Hörmann, Kinga Rigler-Hohenwarter, Rainer Gattringer, Wegene Borena, Dorothee von Laer, Christoph Bock, Andreas Bergthaler.
- **Accession ID:** EPI_ISL_476067 - **Originating Laboratory:** The National Institute of Public Health – **Submiting Laboratory:** State Veterinary Institute Prague and The National Institute of Public Health **- Authors:** Nagy, A; Jirincova, H; Novakova,L; Trnka,D; Vecerova,J.
- **Accession ID:** EPI_ISL_476077 - **Originating Laboratory:** University of Debrecen, Department of Medical Microbiology – **Submiting Laboratory:** National Laboratory of Virology, Szentágothai Research Centre – **Authors:** Endre Gábor Tóth, Balázs Somogyi, Brigitta Zana, Eszter Csoma, Ferenc Jakab, Gábor Kemenesi.
- **Accession ID:** EPI_ISL_476141 - **Originating Laboratory:** Klinisk Mikrobiologi – **Submiting Laboratory:** The Public Health Agency of Sweden **- Authors:** Oskar Karlsson Lindsjo, Maria Lind Karlberg, Mattias Haukland, Reza Advani, Olov Svartstrom, Anna-Malin Linde, Sandra Broddesson, Petra Edquist, Shamam Muradrasoli, Anna Risberg, Karin Tegmark-Wisell.
- **Accession ID:** EPI_ISL_476822 - **Originating Laboratory:** Laboratoire des Fièvres Hémorragiques Virales du Benin – **Submiting Laboratory:** Charité-Universitätsmedizin Berlin **- Authors:** Yadouleton, Anges; Sander Anna-Lena; Moreira-Soto Andres; Drexler, Jan Felix.
- **Accession ID:** EPI_ISL_476823 - **Originating Laboratory:** Laboratoire des Fièvres Hémorragiques Virales du Benin – **Submiting Laboratory:** Charité-Universitätsmedizin Berlin **- Authors:** Yadouleton, Anges; Sander Anna-Lena; Moreira-Soto Andres; Drexler, Jan Felix.
- **Accession ID:** EPI_ISL_476825 - **Originating Laboratory**: Laboratoire des Fièvres Hémorragiques Virales du Benin – **Submiting Laboratory:** Charité-Universitätsmedizin Berlin **- Authors:** Yadouleton, Anges; Sander Anna-Lena; Moreira-Soto Andres; Drexler, Jan Felix.
- **Accession ID:** EPI_ISL_476826- **Originating Laboratory:** Laboratoire des Fièvres Hémorragiques Virales du Benin – **Submiting Laboratory**: Charité-Universitätsmedizin Berlin **- Authors:** Yadouleton, Anges; Sander Anna-Lena; Moreira-Soto Andres; Drexler, Jan Felix.
- **Accession ID:** EPI_ISL_476827 - **Originating Laboratory:** Laboratoire des Fièvres Hémorragiques Virales du Benin – **Submiting Laboratory:** Charité-Universitätsmedizin Berlin **- Authors:** Yadouleton, Anges; Sander Anna-Lena; Moreira-Soto Andres; Drexler, Jan Felix
- **Accession ID:** EPI_ISL_476828 - **Originating Laboratory:** Laboratoire des Fièvres Hémorragiques Virales du Benin – **Submiting Laboratory:** Charité-Universitätsmedizin Berlin **- Authors:** Yadouleton, Anges; Sander Anna-Lena; Moreira-Soto Andres; Drexler, Jan Felix
- **Accession ID:** EPI_ISL_476829 - **Originating Laboratory:** Laboratoire des Fièvres Hémorragiques Virales du Benin – **Submiting Laboratory:** Charité-Universitätsmedizin Berlin **- Authors:** Yadouleton, Anges; Sander Anna-Lena; Moreira-Soto Andres; Drexler, Jan Felix
- **Accession ID:** EPI_ISL_476830 - **Originating Laboratory:** Laboratoire des Fièvres Hémorragiques Virales du Benin – **Submiting Laboratory:** Charité-Universitätsmedizin Berlin **- Authors:** Yadouleton, Anges; Sander Anna-Lena; Moreira-Soto Andres; Drexler, Jan Felix
- **Accession ID:** EPI_ISL_476831 - **Originating Laboratory**: Laboratoire des Fièvres Hémorragiques Virales du Benin – **Submiting Laboratory:** Charité-Universitätsmedizin Berlin **- Authors:** Yadouleton, Anges; Sander Anna-Lena; Moreira-Soto Andres; Drexler, Jan Felix
- **Accession ID:** EPI_ISL_476833 - **Originating Laboratory:** Laboratoire des Fièvres Hémorragiques Virales du Benin – **Submiting Laboratory:** Charité-Universitätsmedizin Berlin **- Authors:** Yadouleton, Anges; Sander Anna-Lena; Moreira-Soto Andres; Drexler, Jan Felix
- **Accession ID:** EPI_ISL_476834 - **Originating Laboratory:** Laboratoire des Fièvres Hémorragiques Virales du Benin – **Submiting Laboratory:** Charité-Universitätsmedizin Berlin **- Authors:** Yadouleton, Anges; Sander Anna-Lena; Moreira-Soto Andres; Drexler, Jan Felix
- **Accession ID:** EPI_ISL_476943 - **Originating Laboratory:** KU Leuven, Rega Institute, Clinical and Epidemiological Virology – **Submiting Laboratory:** KU Leuven, Rega Institute, Clinical and Epidemiological Virology **- Authors:** Tony Wawina-Bokalanga, Joan Marti-Carerras, Bert Vanmechelen, Piet Maes
- **Accession ID:** EPI_ISL_477200 - **Originating Laboratory:** Istituto Zooprofilattico Sperimentale Puglia e Basilicata; – **Submiting Laboratory:** Beaconlab (Bioinformatics, Evolution and Comparative Genomics lab), Dept of Biosciences, University on Milan **- Authors:** Parisi A., Pesole G., Manzari C., Chiara M.
- **Accession ID:** EPI_ISL_477203 - **Originating Laboratory:** Istituto Zooprofilattico Sperimentale Puglia e Basilicata; – **Submiting Laboratory:** Beaconlab (Bioinformatics, Evolution and Comparative Genomics lab), Dept of Biosciences, University on Mila **- Authors:** Parisi A.,Pesole G., Manzari C., Chiara M.
- **Accession ID:** EPI_ISL_479691 - **Originating Laboratory:** Egyptian National Cancer Institute (ENCI) – **Submiting Laboratory:** Egyptian National Cancer Institute (ENCI) **- Authors:** Zekri, Abdel Rahman N, Amer,K.E., Ahmed,O.S., Soliman,H.K., Hafez,M.M., Bahnassy,A.A., Abdelhamid,W., Gad,A., Ali,M., Hassan,W., Samir,M., Raouf,A., Hamdy,M.S., Soliman,M.S., Elsissy,M.H., Elkhateeb,S.M., Ezzelarab,M.H., Abouelhoda, Mohamed
- **Accession ID:** EPI_ISL_479732 - **Originating Laboratory:** Egyptian National Cancer Institute (ENCI) – **Submiting Laboratory:** Egyptian National Cancer Institute (ENCI) **- Authors:** Zekri, Abdel Rahman N, Amer,K.E., Ahmed,O.S., Soliman,H.K., Hafez,M.M., Bahnassy,A.A., Abdelhamid,W., Gad,A., Ali,M., Hassan,W., Samir,M., Raouf,A., Hamdy,M.S., Soliman,M.S., Elsissy,M.H., Elkhateeb,S.M., Ezzelarab,M.H., Abouelhoda, Mohamed
- **Accession ID:** EPI_ISL_480284 - **Originating Laboratory:** Genomic Laboratory (GLAB) (Conjoint lab of Health Directorate of Istanbul and Istanbul Technical University) – **Submiting Laboratory:** Genomic Laboratory (GLAB), Istanbul Technical University **- Authors:** Ilker Karacan, Tugba Kizilboga Akgun, Bugra Agaoglu, Gizem Alkurt, Jale Yildiz, Betsi Köse, Elifnaz Çelik, Arzu Irvem, Yasemin Kendir Demirkol, Ozlem Akgun Dogan, Mehtap Aydın, Levent Doganay, Gizem Dinler Doganay
- **Accession ID:** EPI_ISL_480306 - **Originating Laboratory:** National Reference Laboratory "Influenza and acute respiratory diseases" – **Submiting Laboratory:** NRL-HIV **- Authors:** Ivan Ivanov, Ivailo Alexiev, Ivva Philipova
- **Accession ID:** EPI_ISL_481088 - **Originating Laboratory:** Hospital General Universitario Gregorio Marañón – **Submiting Laboratory:** SeqCOVID-SPAIN consortium/IBV(CSIC) **- Authors:** Laura Pérez-Lago, Marta Herranz, Jon Sicilia, Julia Suárez, Pilar Catalán, Patricia Muñoz, Darío García de Viedma and SeqCOVID-SPAIN consortium
- **Accession ID:** EPI_ISL_481095 - **Originating Laboratory:** Hospital General Universitario Gregorio Marañón – **Submiting Laboratory:** SeqCOVID-SPAIN consortium/IBV(CSIC) **- Authors:** Laura Pérez-Lago, Marta Herranz, Jon Sicilia, Julia Suárez, Pilar Catalán, Patricia Muñoz, Darío García de Viedma and SeqCOVID-SPAIN consortium
- **Accession ID:** EPI_ISL_481695 - **Originating Laboratory:** Department of Virology and Immunology, University of Helsinki and Helsinki University Hospital, Huslab Finland – **Submiting Laboratory:** Department of Virology, Faculty of Medicine, University of Helsinki, Helsinki, Finland **- Authors:** Teemu Smura, Hannimari Kallio-Kokko, Jenni Virtanen, Maija Suvanto, Sari Hannula, Harri Kangas, Pekka Ellonen, Olli Vapalahti
- **Accession ID:** EPI_ISL_481700 - **Originating Laboratory:** Department of Virology and Immunology, University of Helsinki and Helsinki University Hospital, Huslab Finland – **Submiting Laboratory:** Department of Virology, Faculty of Medicine, University of Helsinki, Helsinki, Finland **- Authors:** Teemu Smura, Hannimari Kallio-Kokko, Jenni Virtanen, Maija Suvanto, Sari Hannula, Harri Kangas, Pekka Ellonen, Olli Vapalahti
- **Accession ID:** EPI_ISL_482744 - **Originating Laboratory:** Laboratory Diagnostic, Veterinary Specialized Institute Kraljevo – **Submiting Laboratory**: Laboratory Diagnostic, Veterinary Specialized Institute Kraljevo **- Authors:** Vidanovic,D., Tesovic,B., Banovic Djeri,B., Knezevic,A., Vidanovic,D., Tesovic,B., Banovic Djeri,B., Knezevic,A., Afonso,C.
- **Accession ID:** EPI_ISL_482765 - **Originating Laboratory:** Medical Ain Shams Research Institute (MASRI), Ain Shams University – **Submiting Laboratory:** Medical Ain Shams Research Institute (MASRI), Ain Shams University **- Authors:** Hesham Elghazaly, Sara Hassan Agwa, Ahmad Moustafa, Hala Hafez, Sara Elnakeep, Shaimaa Moustafa, Aya Mohamed, Reham Mamdouh, Ghada Ismael, Ashraf Omar, Osama Mansour, Mahmoud Elmeitini
- **Accession ID:** EPI_ISL_482769 - **Originating Laboratory:** Medical Ain Shams Research Institute (MASRI), Ain Shams University – **Submiting Laboratory:** Medical Ain Shams Research Institute (MASRI), Ain Shams University **- Authors:** Hesham Elghazaly, Sara Hassan Agwa, Ahmad Moustafa, Hala Hafez, Sara Elnakeep, Shaimaa Moustafa, Aya Mohamed, Reham Mamdouh, Ghada Ismael, Ashraf Omar, Osama Mansour, Mahmoud Elmeitini
- **Accession ID:** EPI_ISL_482773 - **Originating Laboratory:** Medical Ain Shams Research Institute (MASRI), Ain Shams University – **Submiting Laboratory**: Medical Ain Shams Research Institute (MASRI), Ain Shams University **- Authors:** Hesham Elghazaly, Sara Hassan Agwa, Ahmad Moustafa, Hala Hafez, Sara Elnakeep, Shaimaa Moustafa, Aya Mohamed, Reham Mamdouh, Ghada Ismael, Ashraf Omar, Osama Mansour, Mahmoud Elmeitini
- **Accession ID:** EPI_ISL_483035 - **Originating Laboratory**: Medical Ain Shams Research Institute (MASRI), Ain Shams University – **Submiting Laboratory:** Medical Ain Shams Research Institute (MASRI), Ain Shams University - **Authors:** Hesham Elghazaly, Sara Hassan Agwa, Ahmad Moustafa, Hala Hafez, Sara Elnakeep, Shaimaa Moustafa, Aya Mohamed, Reham Mamdouh, Ghada Ismael, Ashraf Omar, Osama Mansour, Mahmoud Elmeitini
- **Accession ID:** EPI_ISL_483036 - **Originating Laboratory:** Medical Ain Shams Research Institute (MASRI), Ain Shams University – **Submiting Laboratory:** Medical Ain Shams Research Institute (MASRI), Ain Shams University **- Authors:** Hesham Elghazaly, Sara Hassan Agwa, Ahmad Moustafa, Hala Hafez, Sara Elnakeep, Shaimaa Moustafa, Aya Mohamed, Reham Mamdouh, Ghada Ismael, Ashraf Omar, Osama Mansour, Mahmoud Elmeitini
- **Accession ID:** EPI_ISL_483672 - **Originating Laboratory:** University Hospital Zurich – **Submiting Laboratory:** Department of Biosystems Science and Engineering, ETH Zürich **- Authors:** Christian Beisel, Sarah Nadeau, Ivan Topolsky, Pedro Ferreira, Philipp Jablonski, Susana Posada-Céspedes, Tobias Schär, Ina Nissen, Natascha Santacroce, Elodie Burcklen, Julia Martinez-Gomez, Phil Cheng, Mitch Levesque, Philipp Bosshard, Niko Beerenwinkel, Tanja Stadler
- **Accession ID:** EPI_ISL_484707 - **Originating Laboratory:** Department of Clinical Microbiology – **Submiting Laboratory:** GIGA Medical Genomics **- Authors:** Keith Durkin, Maria Artesi, Sébastien Bontems, Raphaël Boreux, Cécile Meex, Axelle Chaslain, Céline Fombellida-Lopez, Pierrette Melin, Marie-Pierre Hayette, Vincent Bours.
- **Accession ID:** EPI_ISL_485603 - **Originating Laboratory:** Division of Infectious Disease – **Submiting Laboratory:** Steininger Lab **- Authors:** Jakob Thannesberger, Ingeborg Klymiuk, Nicolas Rascovan, Lorenz Schubert, Oliver Robak, Christoph Steininger
- **Accession ID:** EPI_ISL_485813- **Originating Laboratory:** Institut für Virologie und Epidemiologie der Viruskrankheiten, Universitätsklinikum Tübingen – **Submiting Laboratory:** NGS Competence Center Tübingen, Institut für Medizinische Mikrobiologie und Hygiene, Universitätsklinikum Tübingen **- Authors:** Angelov et al.
- **Accession ID:** EPI_ISL_486515 - **Originating Laboratory:** Viollier AG – **Submiting Laboratory:** Department of Biosystems Science and Engineering, ETH Zürich **- Authors:** Christian Beisel, Sarah Nadeau, Ivan Topolsky, Pedro Ferreira, Philipp Jablonski, Susana Posada-Céspedes, Tobias Schär, Ina Nissen, Natascha Santacroce, Elodie Burcklen, Christiane Beckmann, Maurice Redondo, Olivier Kobel, Christoph Noppen, Sophie Seidel, Noemie Santamaria de Souza, Niko Beerenwinkel, Tanja Stadler
- **Accession ID:** EPI_ISL_486537 - **Originating Laboratory:** Viollier AG – **Submiting Laboratory:** Department of Biosystems Science and Engineering, ETH Zürich **- Authors:** Christian Beisel, Sarah Nadeau, Ivan Topolsky, Pedro Ferreira, Philipp Jablonski, Susana Posada-Céspedes, Tobias Schär, Ina Nissen, Natascha Santacroce, Elodie Burcklen, Christiane Beckmann, Maurice Redondo, Olivier Kobel, Christoph Noppen, Sophie Seidel, Noemie Santamaria de Souza, Niko Beerenwinkel, Tanja Stadler
- **Accession ID:** EPI_ISL_486827 - **Originating Laboratory:** Molecular diagnostic laboratory of Federal Budget Institution of Science "Central Research Institute of Epidemiology" of The Federal Service on Customers' Rights Protection and Human Well-being Surveillance – **Submiting Laboratory:** Group of Genomics and Postgenomic Technologies of Central Research Institute of Epidemilology **- Authors:** Speranskaya AS, Kaptelova VV, Valdokhina AV, Bulanenko VP, Samoilov AE, Korneenko EV, Tivanova EV, Shipulina OY, Akimkin VG
- **Accession ID:** EPI_ISL_487370- **Originating Laboratory**: Hellenic Pasteur Institute, National Influenza Reference laboratory of Southern Greece & Unit of Bioinformatics and Applied Genomics – **Submiting Laboratory:** Hellenic Pasteur Institute, National Influenza Reference laboratory of Southern Greece & Unit of Bioinformatics and Applied Genomics - **Authors:** Vasiliki Pogka, Timokratis Karamitros, Athanasios Kossyvakis, Antonios Kalliaropoulos, Horefti Elina, Evangelidou Maria, Androniki Voulgari-Kokota, Aspasia Kontou, Andreas Mentis
- **Accession ID:** EPI_ISL_487381 - **Originating Laboratory:** Hellenic Pasteur Institute, National Influenza Reference laboratory of Southern Greece & Unit of Bioinformatics and Applied Genomics – **Submiting Laboratory:** Hellenic Pasteur Institute, National Influenza Reference laboratory of Southern Greece & Unit of Bioinformatics and Applied Genomics **- Authors:** Vasiliki Pogka, Timokratis Karamitros, Athanasios Kossyvakis, Antonios Kalliaropoulos, Horefti Elina, Evangelidou Maria, Androniki Voulgari-Kokota, Aspasia Kontou, Andreas Mentis
- **Accession ID:** EPI_ISL_489373 - **Originating Laboratory:** Regional Virus Laboratory, Belfast Health and Social Care Trust – **Submiting Laboratory:** Wellcome Sanger Institute for the COVID-19 Genomics UK Consortium **- Authors:** Conall McCaughey, James McKenna, Tanya Curran, Susan Feeney, Alison Watt, Ciara Cox, Mairead Connor, Zoltan Molnar, David Simpson, Derek Fairley; and Alex Alderton, Roberto Amato, Sonia Goncalves, Ewan Harrison, David K. Jackson, Ian Johnston, Dominic Kwiatkowski, Cordelia Langford, John Sillitoe on behalf of the Wellcome Sanger Institute COVID-19 Surveillance Team (http://www.sanger.ac.uk/covid-team)
- **Accession ID:** EPI_ISL_489377 - **Originating Laboratory:** Regional Virus Laboratory, Belfast Health and Social Care Trust – **Submiting Laboratory:** Wellcome Sanger Institute for the COVID-19 Genomics UK Consortium **- Authors:** Conall McCaughey, James McKenna, Tanya Curran, Susan Feeney, Alison Watt, Ciara Cox, Mairead Connor, Zoltan Molnar, David Simpson, Derek Fairley; and Alex Alderton, Roberto Amato, Sonia Goncalves, Ewan Harrison, David K. Jackson, Ian Johnston, Dominic Kwiatkowski, Cordelia Langford, John Sillitoe on behalf of the Wellcome Sanger Institute COVID-19 Surveillance Team (http://www.sanger.ac.uk/covid-team)
- **Accession ID:** EPI_ISL_489961- **Originating Laboratory:** Viollier AG – **Submiting Laboratory:** Department of Biosystems Science and Engineering, ETH Zürich **- Authors:** Christian Beisel, Sarah Nadeau, Ivan Topolsky, Pedro Ferreira, Philipp Jablonski, Susana Posada-Céspedes, Tobias Schär, Ina Nissen, Natascha Santacroce, Elodie Burcklen, Christiane Beckmann, Maurice Redondo, Olivier Kobel, Christoph Noppen, Sophie Seidel, Noemie Santamaria de Souza, Niko Beerenwinkel, Tanja Stadler
- **Accession ID:** EPI_ISL_490348 - **Originating Laboratory:** Liverpool Clinical Laboratories – **Submiting Laboratory:** COVID-19 Genomics UK (COG-UK) Consortium **- Authors**: Sam Haldenby, Anita Lucaci, Steve Paterson, Julian Hiscox, Alistair Darby, M Almsaud, A Alrezaihi, Muhannad Alruwaili, Stuart D Armstrong, Jones Benjamin, Eleanor G Bentley, Anu Chawla, Jordan J Clark, Angela Cowell, Richard Eccles, Isabel García-Dorival, Matthew Gemmell, Alessandro Gerada, PKF Gilmore, Richard Gregory, Ximeng Han, Catherine Hartley, Margaret Hughes, Miren Iturriza-Gomara, James Johnson, L Luu, Jenifer Manson, Charlotte Nelson, Elaine O’Toole, Cassie Olateju, Rebekah Penrice-Randal , Lucille Rainbow, N.P Randle, Trevor Ian Robinson, Parul Sharma, Ghada T Shawli, James P Stewart, Neil Swainston, Ecaterina Vamos, Joanne Watts, Mark Whitehead
- **Accession ID:** EPI_ISL_491078 - **Originating Laboratory:** Suceava County Emergency Hospital – **Submiting Laboratory:** "Stefan cel Mare" University Metagenomics Lab **- Authors:** Lobiuc Andrei et al.
- **Accession ID:** EPI_ISL_491081- **Originating Laboratory:** Suceava County Emergency Hospital – **Submiting Laboratory:** "Stefan cel Mare" University Metagenomics Lab **- Authors:** Lobiuc Andrei et al.
- **Accession ID:** EPI_ISL_491115 - **Originating Laboratory:** Cicin-Sain Lab – **Submiting Laboratory:** Cicin-Sain Lab **- Authors:** M. Zeeshan Chaudhry, Kathrin Eschke, Yeonsu Kim, Luka Cicin-Sain
- **Accession ID:** EPI_ISL_491285 - **Originating Laboratory**: Instituto Gulbenkian de Ciência – **Submiting Laboratory:** Instituto Gulbenkian de Ciência **- Authors:** Susana Ladeiro, João Costa, Cathy Paulino, Joao Sobral, Ricardo Leite
- **Accession ID:** EPI_ISL_492086 - **Originating Laboratory:** Institute for Public Health of the Republic of North Macedonia – **Submiting Laboratory:** Charite Universitatsmedizin Berlin, Institute of Virology **- Authors:** Victor M Corman, Joern Beheim-Schwarzbach, Barbara Mühlemann, Talitha Veith, Julia Schneider, Elizabeta Jancheska, Maja Kuzmanovska, Golubinka Bosevska, Terry Jones, Christian Drosten
- **Accession ID:** EPI_ISL_492995 - **Originating Laboratory:** E. Gulbja Laboratorija – **Submiting Laboratory:** Latvian Biomedical Research and Study Centre **- Authors:** Ivars Silamiķelis, Kaspars Megnis, Monta Ustinova, Ņikita Zrelovs, Vita Rovīte, Mikus Gavars, Dmitrijs Perminovs, Uga Dumpis, Jānis Kloviņš
- **Accession ID:** EPI_ISL_493000 - **Originating Laboratory:** E. Gulbja Laboratorija – **Submiting Laboratory:** Latvian Biomedical Research and Study Centre **- Authors:** Ivars Silamiķelis, Kaspars Megnis, Monta Ustinova, Ņikita Zrelovs, Vita Rovīte, Mikus Gavars, Dmitrijs Perminovs, Uga Dumpis, Jānis Kloviņš
- **Accession ID:** EPI_ISL_493360 - **Originating Laboratory:** Furst Medical Laboratory – **Submiting Laboratory:** Norwegian Institute of Public Health, Department of Virology **- Authors:** Kathrine Stene-Johansen, Kamilla Heddeland Instefjord, Hilde Elshaug, Rasmus Riis Kopperud, Karoline Bragstad, Olav Hungnes
- **Accession ID:** EPI_ISL_493972 - **Originating Laboratory:** Virology Department, Royal Infirmary of Edinburgh, NHS Lothian / School of Biological Sciences, University of Edinburgh / Institute of Genetics and Molecular Medicine, University of Edinburgh – **Submiting Laboratory:** COVID-19 Genomics UK (COG-UK) Consortium **- Authors:** McHugh M, Dewar R, Rooke S, Gallagher M, Balcaza C, O’Toole Á, Scher E, Hill V, McCrone JT, Colquhoun R, Yu X, Jackson B, Rambaut A, Williams TC, Templeton K
- **Accession ID:** EPI_ISL_494347 - **Originating Laboratory:** Originating lab: Wales Specialist Virology Centre Sequencing lab: Pathogen Genomics Unit – **Submiting Laboratory:** COVID-19 Genomics UK (COG-UK) Consortium **- Authors**: Catherine Moore, Johnathan Evans, Laura Gifford, Malorie Perry, Simon Cottrell, Angela Marchbank, Alec Birchley, Alexander Adams, Amy Gaskin, Bree Gatica-Wilcox, Jason Coombes, Joel Southgate, Lauren Gilbert, Lee Graham, Nicole Pacchiarini, Sara Kumziene-Summerhayes, Sarah Taylor, Sophie Jones, Sara Rey, Matthew Bull, Joanne Watkins, Sally Corden, Tom Connor
- **Accession ID:** EPI_ISL_495117 - **Originating Laboratory:** PHE South West Regional Laboratory, National Infection Service – **Submiting Laboratory:** Wellcome Sanger Institute for the COVID-19 Genomics UK Consortium **- Authors:** Stephanie Hutchings, Hannah Pymont, Dr Peter Muir, Barry Vipond, Rich Hopes; and Alex Alderton, Roberto Amato, Sonia Goncalves, Ewan Harrison, David K. Jackson, Ian Johnston, Dominic Kwiatkowski, Cordelia Langford, John Sillitoe on behalf of the Wellcome Sanger Institute COVID-19 Surveillance Team (http://www.sanger.ac.uk/covid-team)
- **Accession ID:** EPI_ISL_495120 - **Originating Laboratory:** PHE South West Regional Laboratory, National Infection Service – **Submiting Laboratory:** Wellcome Sanger Institute for the COVID-19 Genomics UK Consortium **- Authors:** Stephanie Hutchings, Hannah Pymont, Dr Peter Muir, Barry Vipond, Rich Hopes; and Alex Alderton, Roberto Amato, Sonia Goncalves, Ewan Harrison, David K. Jackson, Ian Johnston, Dominic Kwiatkowski, Cordelia Langford, John Sillitoe on behalf of the Wellcome Sanger Institute COVID-19 Surveillance Team (http://www.sanger.ac.uk/covid-team)
- **Accession ID:** EPI_ISL_495452 - **Originating Laboratory:** Kafkas University, Faculty of Medicine, Department of Medical Microbiology – **Submiting Laboratory:** Kafkas University, Faculty of Medicine, Department of Medical Microbiology **- Authors:** Murat Karamese, Didem Ozgur, E. Ediz Tutuncu
- **Accession ID:** EPI_ISL_498815 - **Originating Laboratory**: National Institute of Laboratory Medicine and Referral Center – **Submiting Laboratory:** Genomic Research Lab, BCSIR **- Authors:** Abu Sayeed Mohammad Mahmud, Mohammad Samir Uzzaman, Eshrar Osman, Md. Ahasan Habib, Shahina Akter, Tanjina Akhter Banu, Md. Murshed Hasan Sarkar, Barna Goswami, Iffat Jahan, Md. Saddam Hossain, Tasnim Nafisa, Md. Maruf Ahmed Molla, Mahmuda Yeasmin, Asish Kumar Ghosh, A. K. M. Shamsuzzaman, Sheikh Md. Selim Al Din, Utpal Chandra Ray, Salek Ahmed Sajib, Md. Salim Khan
- **Accession ID:** EPI_ISL_504179 - **Originating Laboratory:** National Institute of Laboratory Medicine and Referral Center – **Submiting Laboratory:** Genomic Research Lab, BCSIR **- Authors:** Abu Sayeed Mohammad Mahmud, Mohammad Samir Uzzaman, Eshrar Osman, Md. Ahasan Habib, Shahina Akter, Tanjina Akhter Banu, Md. Murshed Hasan Sarkar, Barna Goswami, Iffat Jahan, Md. Saddam Hossain, Tarannum Taznin, Tasnim Nafisa, Md. Maruf Ahmed Molla, Mahmuda Yeasmin, Asish Kumar Ghosh, A. K. M. Shamsuzzaman, Sheikh Md. Selim Al Din, Utpal Chandra Ray, Salek Ahmed Sajib, Md. Salim Khan
- **Accession ID:** EPI_ISL_504181 - **Originating Laboratory:** National Institute of Laboratory Medicine and Referral Center – **Submiting Laboratory:** Genomic Research Lab, BCSIR **- Authors:** Abu Sayeed Mohammad Mahmud, Mohammad Samir Uzzaman, Eshrar Osman, Md. Ahasan Habib, Shahina Akter, Tanjina Akhter Banu, Md. Murshed Hasan Sarkar, Barna Goswami, Iffat Jahan, Md. Saddam Hossain, Tarannum Taznin, Tasnim Nafisa, Md. Maruf Ahmed Molla, Mahmuda Yeasmin, Asish Kumar Ghosh, A. K. M. Shamsuzzaman, Sheikh Md. Selim Al Din, Utpal Chandra Ray, Salek Ahmed Sajib, Md. Salim Khan
- **Accession ID:** EPI_ISL_504183 - **Originating Laboratory:** National Institute of Laboratory Medicine and Referral Center – **Submiting Laboratory:** Genomic Research Lab, BCSIR **- Authors:** Abu Sayeed Mohammad Mahmud, Mohammad Samir Uzzaman, Eshrar Osman, Md. Ahasan Habib, Shahina Akter, Tanjina Akhter Banu, Md. Murshed Hasan Sarkar, Barna Goswami, Iffat Jahan, Md. Saddam Hossain, Tarannum Taznin, Tasnim Nafisa, Md. Maruf Ahmed Molla, Mahmuda Yeasmin, Asish Kumar Ghosh, A. K. M. Shamsuzzaman, Sheikh Md. Selim Al Din, Utpal Chandra Ray, Salek Ahmed Sajib, Md. Salim Khan
- **Accession ID:** EPI_ISL_512873 - **Originating Laboratory:** Centre Pasteur of Cameroun – **Submiting Laboratory:** Virology Service, Centre Pasteur of Cameroun **- Authors:** Richard Njouom and Serge Alain SADEUH-Mba
- **Accession ID:** EPI_ISL_513597 - **Originating Laboratory:** Viral Respiratory Lab, National Institute for Biomedical Research (INRB) – **Submiting Laboratory:** Pathogen Sequencing Lab, National Institute for Biomedical Research (INRB) **- Authors:** Placide Mbala-Kingebeni, Edith Nkwembe, Eddy Kinganda-Lusamaki, Amuri Aziza, Francisca Muyembe Mawete, Emmanuel Lokilo Lofiko, Catherine Pratt, Matthias Pauthner, Josh Quick, Allison Black, James Hadfield, Trevor Bedford, Ian Goodfellow, Andrew Rambaut, Nick Loman, Kristian Andersen, Michael Wiley, Steve Ahuka-Mundeke, Jean-Jacques Muyembe Tamfum
- **Accession ID:** EPI_ISL_526975- **Originating Laboratory:** Biological prevention, army – **Submiting Laboratory:** Biological prevention, army **- Authors:** Seadawy, M.G., Gad, A.F., Harty, B.E., Elhosieny, M.F., Shamel, M.D.
- **Accession ID:** EPI_ISL_527511 - **Originating Laboratory:** Viral Respiratory Lab, National Institute for Biomedical Research (INRB) – **Submiting Laboratory:** Pathogen Sequencing Lab, National Institute for Biomedical Research (INRB) **- Authors:** Placide Mbala-Kingebeni, Edith Nkwembe, Eddy Kinganda-Lusamaki, Amuri Aziza, Francisca Muyembe Mawete, Emmanuel Lokilo Lofiko, Catherine Pratt, Matthias Pauthner, Josh Quick, Allison Black, James Hadfield, Trevor Bedford, Ian Goodfellow, Andrew Rambaut, Nick Loman, Kristian Andersen, Michael Wiley, Steve Ahuka-Mundeke, Jean-Jacques Muyembe Tamfum
- **Accession ID:** EPI_ISL_527523 - **Originating Laboratory:** Viral Respiratory Lab, National Institute for Biomedical Research (INRB) – **Submiting Laboratory:** Pathogen Sequencing Lab, National Institute for Biomedical Research (INRB) **- Authors:** Placide Mbala-Kingebeni, Edith Nkwembe, Eddy Kinganda-Lusamaki, Amuri Aziza, Francisca Muyembe Mawete, Emmanuel Lokilo Lofiko, Catherine Pratt, Matthias Pauthner, Josh Quick, Allison Black, James Hadfield, Trevor Bedford, Ian Goodfellow, Andrew Rambaut, Nick Loman, Kristian Andersen, Michael Wiley, Steve Ahuka-Mundeke, Jean-Jacques Muyembe Tamfum
- **Accession ID:** EPI_ISL_527543 - **Originating Laboratory:** Viral Respiratory Lab, National Institute for Biomedical Research (INRB) – **Submiting Laboratory:** Pathogen Sequencing Lab, National Institute for Biomedical Research (INRB) **- Authors:** Placide Mbala-Kingebeni, Edith Nkwembe, Eddy Kinganda-Lusamaki, Amuri Aziza, Francisca Muyembe Mawete, Emmanuel Lokilo Lofiko, Catherine Pratt, Matthias Pauthner, Josh Quick, Allison Black, James Hadfield, Trevor Bedford, Ian Goodfellow, Andrew Rambaut, Nick Loman, Kristian Andersen, Michael Wiley, Steve Ahuka-Mundeke, Jean-Jacques Muyembe Tamfum
- **Accession ID:** EPI_ISL_527566- **Originating Laboratory:** Viral Respiratory Lab, National Institute for Biomedical Research (INRB) – **Submiting Laboratory:** Pathogen Sequencing Lab, National Institute for Biomedical Research (INRB) **- Authors:** Placide Mbala-Kingebeni, Edith Nkwembe, Eddy Kinganda-Lusamaki, Amuri Aziza, Francisca Muyembe Mawete, Emmanuel Lokilo Lofiko, Catherine Pratt, Matthias Pauthner, Josh Quick, Allison Black, James Hadfield, Trevor Bedford, Ian Goodfellow, Andrew Rambaut, Nick Loman, Kristian Andersen, Michael Wiley, Steve Ahuka-Mundeke, Jean-Jacques Muyembe Tamfum
- **Accession ID:** EPI_ISL_527571 - **Originating Laboratory:** Viral Respiratory Lab, National Institute for Biomedical Research (INRB) – **Submiting Laboratory:** Pathogen Sequencing Lab, National Institute for Biomedical Research (INRB) **- Authors:** Placide Mbala-Kingebeni, Edith Nkwembe, Eddy Kinganda-Lusamaki, Amuri Aziza, Francisca Muyembe Mawete, Emmanuel Lokilo Lofiko, Catherine Pratt, Matthias Pauthner, Josh Quick, Allison Black, James Hadfield, Trevor Bedford, Ian Goodfellow, Andrew Rambaut, Nick Loman, Kristian Andersen, Michael Wiley, Steve Ahuka-Mundeke, Jean-Jacques Muyembe Tamfum
- **Accession ID:** EPI_ISL_529032 - **Originating Laboratory:** Central Molecular Microbiology Laboratory and Next Generation Sequencing Reference Laboratory, Clinical and Chemical Pathology Department, Faculty of Medicine, CAIRO UNIVERSITY – **Submiting Laboratory:** Next Generation Sequencing Reference Laboratory, Faculty of Medicine, CAIRO UNIVERSITY and The Center for Genome and Microbiome Research, Faculty of Pharmacy, CAIRO UNIVERSITY **- Authors:** May Sherif Soliman, May Abdelfattah, Ramy Karam Aziz
- **Accession ID:** EPI_ISL_529145 - **Originating Laboratory:** Egyptian National Cancer Institute (ENCI) – **Submiting Laboratory:** Egyptian National Cancer Institute (ENCI) **- Authors:** Zekri, Abdel Rahman N., Amer,K.E., Ahmed,O.S., Soliman,H.K., Ali,M.A., Hassan,W.A., Mahmoud,A.A., Khattab,A.A., Hafez,M.M., Abouelhoda, Mohamed
- **Accession ID:** EPI_ISL_539573 - **Originating Laboratory:** Centre de Recherches Medicales de Lambarene (CERMEL) – **Submiting Laboratory:** Department of Emerging Infectious Diseases, Institute of Tropical Medicine, Nagasaki University **- Authors:** Haruka Abe, Yuri Ushijima, Rodrigue Bikangui, Akim A. Adegnika, Bertrand Lell, Jiro Yasuda
- **Accession ID:** EPI_ISL_539574 - **Originating Laboratory:** Centre de Recherches Medicales de Lambarene (CERMEL) – **Submiting Laboratory:** Department of Emerging Infectious Diseases, Institute of Tropical Medicine, Nagasaki University **- Authors**: Haruka Abe, Yuri Ushijima, Rodrigue Bikangui, Akim A. Adegnika, Bertrand Lell, Jiro Yasuda
- **Accession ID:** EPI_ISL_539575 - **Originating Laboratory:** Centre de Recherches Medicales de Lambarene (CERMEL) – **Submiting Laboratory:** Department of Emerging Infectious Diseases, Institute of Tropical Medicine, Nagasaki University **- Authors:** Haruka Abe, Yuri Ushijima, Rodrigue Bikangui, Akim A. Adegnika, Bertrand Lell, Jiro Yasuda
- **Accession ID:** EPI_ISL_539576 - **Originating Laboratory:** Centre de Recherches Medicales de Lambarene (CERMEL) – **Submiting Laboratory:** Department of Emerging Infectious Diseases, Institute of Tropical Medicine, Nagasaki University **- Authors:** Haruka Abe, Yuri Ushijima, Rodrigue Bikangui, Akim A. Adegnika, Bertrand Lell, Jiro Yasuda
- **Accession ID:** EPI_ISL_428855 - **Originating Laboratory:** MRCG at LSHTM Geomics lab – **Submiting Laboratory:** MRCG at LSHTM Geomics lab **- Authors:** Sesay et al
- **Accession ID:** EPI_ISL_428856 - **Originating Laboratory:** MRCG at LSHTM Genomics Lab – **Submiting Laboratory:** MRCG at LSHTM Genomics Lab **- Authors:** Sesay et al
- **Accession ID:** EPI_ISL_471158 - **Originating Laboratory:** MRCG at LSHTM Genomics lab – **Submiting Laboratory:** MRCG at LSHTM Genomics lab **- Authors:** Sesay et al
- **Accession ID:** EPI_ISL_471160 - **Originating Laboratory:** MRCG at LSHTM Genomics lab – **Submiting Laboratory:** MRCG at LSHTM Genomics lab **- Authors:** Sesay et al
- **Accession ID:** EPI_ISL_471166 - **Originating Laboratory:** MRCG at LSHTM Genomics lab – **Submiting Laboratory:** MRCG at LSHTM Genomics lab **- Authors:** Sesay et al
- **Accession ID:** EPI_ISL_422384 - **Originating Laboratory:** NMIMR, Department of Virology – **Submiting Laboratory:** WACCBIP, University of Ghana **- Authors:** Joyce M. Ngoi, Bright Adu, Collins M. Morang’a, Selassie Kumordjie, Miriam Eshun, Linda Boatemaa, Vanessa Magnussen, Erasmus Kotey, Fred Tei-Maya, Dominic S. Y. Amuzu, Peter Quashie, Augustina Arjarquah, Ivy Asante, Evelyn Bonney, George B. Kyei, Kofi Bonney, Abraham Kwabena Anang, Gordon A. Awandare, William Ampofo
- **Accession ID:** EPI_ISL_422390 - **Originating Laboratory:** NMIMR, Department of Virology – **Submiting Laboratory:** WACCBIP, University of Ghana **- Authors:** Joyce M. Ngoi, Bright Adu, Collins M. Morang’a, Selassie Kumordjie, Miriam Eshun, Linda Boatemaa, Vanessa Magnussen, Erasmus Kotey, Fred Tei-Maya, Dominic S. Y. Amuzu, Peter Quashie, Augustina Arjarquah, Ivy Asante, Evelyn Bonney, George B. Kyei, Kofi Bonney, Abraham Kwabena Anang, Gordon A. Awandare, William Ampofo
- **Accession ID:** EPI_ISL_422394 - **Originating Laboratory:** NMIMR, Department of Virology – **Submiting Laboratory:** WACCBIP, University of Ghana **- Authors:** Joyce M. Ngoi, Bright Adu, Collins M. Morang’a, Selassie Kumordjie, Miriam Eshun, Linda Boatemaa, Vanessa Magnussen, Erasmus Kotey, Fred Tei-Maya, Dominic S. Y. Amuzu, Peter Quashie, Augustina Arjarquah, Ivy Asante, Evelyn Bonney, George B. Kyei, Kofi Bonney, Abraham Kwabena Anang, Gordon A. Awandare, William Ampofo
- **Accession ID:** EPI_ISL_422397 - **Originating Laboratory:** NMIMR, Department of Virology – **Submiting Laboratory:** WACCBIP, University of Ghana **- Authors:** Joyce M. Ngoi, Bright Adu, Collins M. Morang’a, Selassie Kumordjie, Miriam Eshun, Linda Boatemaa, Vanessa Magnussen, Erasmus Kotey, Fred Tei-Maya, Dominic S. Y. Amuzu, Peter Quashie, Augustina Arjarquah, Ivy Asante, Evelyn Bonney, George B. Kyei, Kofi Bonney, Abraham Kwabena Anang, Gordon A. Awandare, William Ampofo
- **Accession ID:** EPI_ISL_422398 - **Originating Laboratory:** NMIMR, Department of Virology – **Submiting Laboratory:** WACCBIP, University of Ghana **- Authors:** Joyce M. Ngoi, Bright Adu, Collins M. Morang’a, Selassie Kumordjie, Miriam Eshun, Linda Boatemaa, Vanessa Magnussen, Erasmus Kotey, Fred Tei-Maya, Dominic S. Y. Amuzu, Peter Quashie, Augustina Arjarquah, Ivy Asante, Evelyn Bonney, George B. Kyei, Kofi Bonney, Abraham Kwabena Anang, Gordon A. Awandare, William Ampofo
- **Accession ID:** EPI_ISL_422400 - **Originating Laboratory:** NMIMR, Department of Virology – **Submiting Laboratory:** WACCBIP, University of Ghana **- Authors:** Joyce M. Ngoi, Bright Adu, Collins M. Morang’a, Selassie Kumordjie, Miriam Eshun, Linda Boatemaa, Vanessa Magnussen, Erasmus Kotey, Fred Tei-Maya, Dominic S. Y. Amuzu, Peter Quashie, Augustina Arjarquah, Ivy Asante, Evelyn Bonney, George B. Kyei, Kofi Bonney, Abraham Kwabena Anang, Gordon A. Awandare, William Ampofo
- **Accession ID:** EPI_ISL_422401 - **Originating Laboratory:** NMIMR, Department of Virology – **Submiting Laboratory:** WACCBIP, University of Ghana **- Authors:** Joyce M. Ngoi, Bright Adu, Collins M. Morang’a, Selassie Kumordjie, Miriam Eshun, Linda Boatemaa, Vanessa Magnussen, Erasmus Kotey, Fred Tei-Maya, Dominic S. Y. Amuzu, Peter Quashie, Augustina Arjarquah, Ivy Asante, Evelyn Bonney, George B. Kyei, Kofi Bonney, Abraham Kwabena Anang, Gordon A. Awandare, William Ampofo
- **Accession ID:** EPI_ISL_422404 - **Originating Laboratory:** NMIMR, Department of Virology – **Submiting Laboratory:** WACCBIP, University of Ghana **- Authors:** Joyce M. Ngoi, Bright Adu, Collins M. Morang’a, Selassie Kumordjie, Miriam Eshun, Linda Boatemaa, Vanessa Magnussen, Erasmus Kotey, Fred Tei-Maya, Dominic S. Y. Amuzu, Peter Quashie, Augustina Arjarquah, Ivy Asante, Evelyn Bonney, George B. Kyei, Kofi Bonney, Abraham Kwabena Anang, Gordon A. Awandare, William Ampofo
- **Accession ID:** EPI_ISL_422405 - **Originating Laboratory:** NMIMR, Department of Virology – **Submiting Laboratory:** NMIMR, Department of Virology **- Authors:** Joyce M. Ngoi, Bright Adu, Collins M. Morang’a, Selassie Kumordjie, Miriam Eshun, Linda Boatemaa, Vanessa Magnussen, Erasmus Kotey, Fred Tei-Maya, Dominic S. Y. Amuzu, Peter Quashie, Augustina Arjarquah, Ivy Asante, Evelyn Bonney, George B. Kyei, Kofi Bonney, Abraham Kwabena Anang, Gordon A. Awandare, William Ampofo
- **Accession ID:** EPI_ISL_457827 - **Originating Laboratory:** National Public Health Laboratory – **Submiting Laboratory:** KEMRI-Wellcome Trust Research Programme/KEMRI-CGMR-C Kilifi **- Authors:** Githinji G. et al 2020
- **Accession ID:** EPI_ISL_457829 - **Originating Laboratory**: National Public Health Laboratory – **Submiting Laboratory:** KEMRI-Wellcome Trust Research Programme/KEMRI-CGMR-C Kilifi **- Authors:** Githinji G. et al 2020
- **Accession ID:** EPI_ISL_457833 - **Originating Laboratory:** National Public Health Laboratory – **Submiting Laboratory:** KEMRI-Wellcome Trust Research Programme/KEMRI-CGMR-C Kilifi **- Authors:** Githinji G. et al 2020
- **Accession ID:** EPI_ISL_457867 - **Originating Laboratory:** KEMRI-CGMR-C – **Submiting** Laboratory: KEMRI-Wellcome Trust Research Programme/KEMRI-CGMR-C Kilifi - Authors: Githinji G. et al 2020
- **Accession ID:** EPI_ISL_457906 - **Originating Laboratory:** KEMRI-CGMR-C – **Submiting Laboratory:** KEMRI-Wellcome Trust Research Programme/KEMRI-CGMR-C Kilifi **- Authors:** Githinji G. et al 2020
- **Accession ID:** EPI_ISL_457920 - **Originating Laboratory:** KEMRI-CGMR-C – **Submiting Laboratory**: KEMRI-Wellcome Trust Research Programme/KEMRI-CGMR-C Kilifi **- Authors:** Githinji G. et al 2020
- **Accession ID:** EPI_ISL_457934 - **Originating Laboratory:** KEMRI-Centre for Virus Research – **Submiting Laboratory:** KEMRI-Wellcome Trust Research Programme/KEMRI-CGMR-C Kilifi **- Authors:** Githinji G. et al 2020
- **Accession ID:** EPI_ISL_508862 - **Originating Laboratory:** Virology Unit, Institut Pasteur de Madagascar – **Submiting Laboratory:** Virology Unit, Institut Pasteur de Madagascar **- Authors:** Christian Ranaivoson, Cara Brook, Norosoa Razanajatovo, Vida Ahyong, Tsiry Randriambolamanantsoa, Michelle Tan, Vololoniaina Raharinosy, Helisoa Razafimanjato, Cristina M. Tato, Joseph L. DeRisi, Soa Fy Andriamandimby, Jean-Michel Heraud
- **Accession ID:** EPI_ISL_487446 - **Originating Laboratory:** CICM-Mali – **Submiting Laboratory:** Bundeswehr Institut of Microbiology **- Authors**: Kouriba, Dürr, Sangaré, Rehn, Traoré, Bestehorn-Willmann, Walter, Quedraogo, Zimmermann, Maiga, Heitzer, Sogodogo, Antwerpen, Wölfel
- **Accession ID:** EPI_ISL_487447 - **Originating Laboratory:** CICM-Mali – **Submiting Laboratory:** Bundeswehr Institut of Microbiology **- Authors**: Kouriba, Dürr, Sangaré, Rehn, Traoré, Bestehorn-Willmann, Walter, Quedraogo, Zimmermann, Maiga, Heitzer, Sogodogo, Antwerpen, Wölfel
- **Accession ID:** EPI_ISL_487449 - **Originating Laboratory:** CICM-Mali – **Submiting Laboratory:** Bundeswehr Institut of Microbiology **- Authors:** Kouriba, Dürr, Sangaré, Rehn, Traoré, Bestehorn-Willmann, Walter, Quedraogo, Zimmermann, Maiga, Heitzer, Sogodogo, Antwerpen, Wölfel
- **Accession ID:** EPI_ISL_487450 - **Originating Laboratory:** CICM-Mali – **Submiting Laboratory:** Bundeswehr Institut of Microbiology **- Authors:** Kouriba, Dürr, Sangaré, Rehn, Traoré, Bestehorn-Willmann, Walter, Quedraogo, Zimmermann, Maiga, Heitzer, Sogodogo, Antwerpen, Wölfel
- **Accession ID:** EPI_ISL_487451 - **Originating Laboratory:** CICM-Mali – **Submiting Laboratory:** Bundeswehr Institut of Microbiology **- Authors**: Kouriba, Dürr, Sangaré, Rehn, Traoré, Bestehorn-Willmann, Walter, Quedraogo, Zimmermann, Maiga, Heitzer, Sogodogo, Antwerpen, Wölfel
- **Accession ID:** EPI_ISL_487452 - **Originating Laboratory:** CICM-Mali – **Submiting Laboratory:** Bundeswehr Institut of Microbiology **- Authors**: Kouriba, Dürr, Sangaré, Rehn, Traoré, Bestehorn-Willmann, Walter, Quedraogo, Zimmermann, Maiga, Heitzer, Sogodogo, Antwerpen, Wölfel
- **Accession ID:** EPI_ISL_487453 - **Originating Laboratory:** CICM-Mali – **Submiting Laboratory:** Bundeswehr Institut of Microbiology **- Authors:** Kouriba, Dürr, Sangaré, Rehn, Traoré, Bestehorn-Willmann, Walter, Quedraogo, Zimmermann, Maiga, Heitzer, Sogodogo, Antwerpen, Wölfel
- **Accession ID:** EPI_ISL_487457 - **Originating Laboratory:** CICM-Mali – **Submiting Laboratory:** Bundeswehr Institut of Microbiology **- Authors:** Kouriba, Dürr, Sangaré, Rehn, Traoré, Bestehorn-Willmann, Walter, Quedraogo, Zimmermann, Maiga, Heitzer, Sogodogo, Antwerpen, Wölfel
- **Accession ID:** EPI_ISL_487459 - **Originating Laboratory:** CICM-Mali – **Submiting Laboratory:** Bundeswehr Institut of Microbiology **- Authors:** Kouriba, Dürr, Sangaré, Rehn, Traoré, Bestehorn-Willmann, Walter, Quedraogo, Zimmermann, Maiga, Heitzer, Sogodogo, Antwerpen, Wölfel
- **Accession ID:** EPI_ISL_487460 - **Originating Laboratory:** CICM-Mali – **Submiting Laboratory:** Bundeswehr Institut of Microbiology **- Authors:** Kouriba, Dürr, Sangaré, Rehn, Traoré, Bestehorn-Willmann, Walter, Quedraogo, Zimmermann, Maiga, Heitzer, Sogodogo, Antwerpen, Wölfel
- **Accession ID:** EPI_ISL_487462 - **Originating Laboratory:** CICM-Mali – **Submiting Laboratory:** Bundeswehr Institut of Microbiology **- Authors:** Kouriba, Dürr, Sangaré, Rehn, Traoré, Bestehorn-Willmann, Walter, Quedraogo, Zimmermann, Maiga, Heitzer, Sogodogo, Antwerpen, Wölfel
- **Accession ID:** EPI_ISL_487463 - **Originating Laboratory:** CICM-Mali – **Submiting Laboratory:** Bundeswehr Institut of Microbiology **- Authors:** Kouriba, Dürr, Sangaré, Rehn, Traoré, Bestehorn-Willmann, Walter, Quedraogo, Zimmermann, Maiga, Heitzer, Sogodogo, Antwerpen, Wölfel
- **Accession ID:** EPI_ISL_487464 - **Originating Laboratory:** CICM-Mali – **Submiting Laboratory:** Bundeswehr Institut of Microbiology **- Authors:** Kouriba, Dürr, Sangaré, Rehn, Traoré, Bestehorn-Willmann, Walter, Quedraogo, Zimmermann, Maiga, Heitzer, Sogodogo, Antwerpen, Wölfel
- **Accession ID:** EPI_ISL_487465 - **Originating Laboratory:** CICM-Mali – **Submiting Laboratory:** Bundeswehr Institut of Microbiology **- Authors:** Kouriba, Dürr, Sangaré, Rehn, Traoré, Bestehorn-Willmann, Walter, Quedraogo, Zimmermann, Maiga, Heitzer, Sogodogo, Antwerpen, Wölfel
- **Accession ID:** EPI_ISL_487466 - **Originating Laboratory**: CICM-Mali – **Submiting Laboratory:** Bundeswehr Institut of Microbiology **- Authors:** Kouriba, Dürr, Sangaré, Rehn, Traoré, Bestehorn-Willmann, Walter, Quedraogo, Zimmermann, Maiga, Heitzer, Sogodogo, Antwerpen, Wölfel
- **Accession ID:** EPI_ISL_451400 - **Originating Laboratory**: Laboratoire de Recherche et d'Analyse Médicale de la Gendarmerie Royale – **Submiting Laboratory:** Laboratoire de Recherche et d'Analyse Médicale de la Gendarmerie Royale **- Authors:** Sanaâ LEMRISS, Amal SOUIRI, Saâd EL KABBAJ
- **Accession ID:** EPI_ISL_458150 - **Originating Laboratory:** ANOUAL – **Submiting Laboratory:** ANOUAL **- Authors:** Jouali Farah, El Ansari Fatima Zahra, Marchoudi Nabila, Kasmi Yassine, Chenaoui Mohamed, El Aliani Aissam, Benhida Rachid, Azami Nawfel, Kitane Driss Lahlou, Loukman Salma, Fekkak Jamal
- **Accession ID:** EPI_ISL_459965 - **Originating Laboratory:** Institut Pasteur du Maroc – **Submiting Laboratory:** Institut Pasteur du Maroc **- Authors**: Marion Barbet, Sylvie Behillil, Méline Bizard, Angela Brisebarre, Camille Capel, Etienne Simon-Lorière, Vincent Enouf, Maud Vanpeene, Sylvie van der Werf, Latifa Anga, Abdellah Faouzi, Anass Abbad, Mjid Eloualid, Jalal Nourlil, Anderrahmane Maaroufi
- **Accession ID:** EPI_ISL_459982 - **Originating Laboratory:** Institut Pasteur du Maroc – **Submiting Laboratory**: Institut Pasteur du Maroc **- Authors:** Marion Barbet, Sylvie Behillil, Méline Bizard, Angela Brisebarre, Camille Capel, Etienne Simon-Lorière, Vincent Enouf, Maud Vanpeene, Sylvie van der Werf, Latifa Anga, Abdellah Faouzi, Anass Abbad, Mjid Eloualid, Jalal Nourlil, Anderrahmane Maaroufi
- **Accession ID:** EPI_ISL_459983 - **Originating Laboratory:** Institut Pasteur du Maroc – **Submiting Laboratory:** Institut Pasteur du Maroc **- Authors:** Marion Barbet, Sylvie Behillil, Méline Bizard, Angela Brisebarre, Camille Capel, Etienne Simon-Lorière, Vincent Enouf, Maud Vanpeene, Sylvie van der Werf, Latifa Anga, Abdellah Faouzi, Anass Abbad, Mjid Eloualid, Jalal Nourlil, Anderrahmane Maaroufi
- **Accession ID:** EPI_ISL_469053 - **Originating Laboratory:** LNR National Reference Laboratory, Mohammed VI University of Health Sciences – **Submiting Laboratory:** Medical Biotechnology Laboratory, Rabat Medical and Pharmacy School, Mohammed The Vth University in Rabat **- Authors:** Meriem LAAMARTI, Souad KARTTI, Rokaia LAAMRTI , M.W. CHEMAO-ELFIHRI, Loubna ALLAM, Mouna OUADGHIRI, Imane SMYEJ, Jalila RAHOUI, Houda BENRAHMA, Jalil El Atar, Idrissa Diawara, Rachid EL JAOUDI, Laila SBABOU, Chakib NEJJARI, Saaid AMZAZI, Rachid MENTAG, Lahcen BELYAMANI and Azeddine IBRAHIMI
- **Accession ID:** EPI_ISL_471459 - **Originating Laboratory:** Centre de Virologie des Maladies Tropicales – **Submiting Laboratory:** Functional Genomic Platform/Service Analyses Biologique/UATRS/ Centre National Pour la Recherche Scientifique Et Technique (CNRST) **- Authors:** Hicham ANNAZ, Elmostafa EL FAHIME, Marouane MELLOUL, Yassine AKHOUAD, Mly Abdelaziz ELALAOUI, Ahmed REGGAD, Sanaa ALAOUI-Amine , Rachid ABI,  Rida TAGAJDID, Zhor KASMY, Safaa ELKORCHI, Nadia TOUIL, Farida HILALI, Abdelkader LAATIRIS , Abdelillah LARAQUI, Tahra BAJJOU , Yassine SEKHSOKH , Idriss-Amine LAHLOU,
- **Accession ID:** EPI_ISL_482738 - **Originating Laboratory:** LNR National Reference Laboratory, Mohammed VI University of Health Sciences – **Submiting Laboratory:** Medical Biotechnology Laboratory, Rabat Medical and Pharmacy School, Mohammed The Vth University in Rabat **- Authors:** Meriem LAAMARTI, Souad KARTTI, Rokia LAAMARTI , M.W. CHEMAO-ELFIHRI, Loubna ALLAM, Mouna OUADGHIRI, Imane SMYEJ, Jalila RAHOUI, Houda BENRAHMA, Jalil El ATAR, Idrissa DIAWARA, Rachid EL JAOUDI, Laila SBABOU, Chakib NEJJARI, Saaid AMZAZI, Rachid MENTAG, Lahcen BELYAMANI and Azeddine IBRAHIMI
- **Accession ID:** EPI_ISL_413550 - **Originating Laboratory:** Centre for Human and Zoonotic Virology (CHAZVY), College of Medicine University of Lagos/Lagos University Teaching Hospital (LUTH), part of the Laboratory Network of the Nigeria Centre for Disease Control (NCDC) – **Submiting Laboratory:** African Centre of Excellence for Genomics of Infectious Diseases (ACEGID), Redeemer's University, Ede, Osun State, Nigeria **- Authors:** Oluniyi P.E., Ajogbasile F.V., Kayode A., Oguzie J., Folarin O.A., Ihekweazu C. Happi C.T.
- **Accession ID:** EPI_ISL_455419 - **Originating Laboratory:** Nigeria Centre for Disease Control (NCDC) – **Submiting Laboratory:** African Centre of Excellence for Genomics of Infectious Diseases (ACEGID), Redeemer's University, Ede, Osun State, Nigeria **- Authors:** Oluniyi P.E., Ajogbasile F.V., Kayode A., Oguzie J., Olawoye I., Uwanibe J., Olumade T., Folarin O.A., Ihekweazu C., Happi C.T.
- **Accession ID:** EPI_ISL_455423 - **Originating Laboratory:** Nigeria Centre for Disease Control (NCDC) – **Submiting Laboratory:** African Centre of Excellence for Genomics of Infectious Diseases (ACEGID), Redeemer's University, Ede, Osun State, Nigeria **- Authors:** Oluniyi P.E., Ajogbasile F.V., Kayode A., Oguzie J., Olawoye I., Uwanibe J., Olumade T., Folarin O.A., Ihekweazu C., Happi C.T.
- **Accession ID:** EPI_ISL_455429 - **Originating Laboratory:** Nigeria Centre for Disease Control (NCDC) – **Submiting Laboratory:** African Centre of Excellence for Genomics of Infectious Diseases (ACEGID), Redeemer's University, Ede, Osun State, Nigeria **- Authors:** Oluniyi P.E., Ajogbasile F.V., Kayode A., Oguzie J., Olawoye I., Uwanibe J., Olumade T., Folarin O.A., Ihekweazu C., Happi C.T.
- **Accession ID:** EPI_ISL_455431 - **Originating Laboratory:** Nigeria Centre for Disease Control (NCDC) – **Submiting Laboratory:** African Centre of Excellence for Genomics of Infectious Diseases (ACEGID), Redeemer's University, Ede, Osun State, Nigeria **- Authors:** Oluniyi P.E., Ajogbasile F.V., Kayode A., Oguzie J., Olawoye I., Uwanibe J., Olumade T., Folarin O.A., Ihekweazu C., Happi C.T.
- **Accession ID:** EPI_ISL_487087 - **Originating Laboratory:** Nigeria Centre for Disease Control (NCDC) – **Submiting Laboratory:** African Centre of Excellence for Genomics of Infectious Diseases (ACEGID), Redeemer's University, Ede, Osun State, Nigeria **- Authors:** Oluniyi P.E., Ajogbasile F.V., Kayode A., Oguzie J., Olawoye I., Uwanibe J., Olumade T., Folarin O.A., Ihekweazu C., Happi C.T.
- **Accession ID:** EPI_ISL_487090 - **Originating Laboratory:** Nigeria Centre for Disease Control (NCDC) – **Submiting Laboratory:** African Centre of Excellence for Genomics of Infectious Diseases (ACEGID), Redeemer's University, Ede, Osun State, Nigeria **- Authors:** Oluniyi P.E., Ajogbasile F.V., Kayode A., Oguzie J., Olawoye I., Uwanibe J., Olumade T., Folarin O.A., Ihekweazu C., Happi C.T.
- **Accession ID:** EPI_ISL_487092 - **Originating Laboratory:** Nigeria Centre for Disease Control (NCDC) – **Submiting Laboratory:** African Centre of Excellence for Genomics of Infectious Diseases (ACEGID), Redeemer's University, Ede, Osun State, Nigeria **- Authors:** Oluniyi P.E., Ajogbasile F.V., Kayode A., Oguzie J., Olawoye I., Uwanibe J., Olumade T., Folarin O.A., Ihekweazu C., Happi C.T.
- **Accession ID:** EPI_ISL_487095 - **Originating Laboratory:** Nigeria Centre for Disease Control (NCDC) – **Submiting Laboratory:** African Centre of Excellence for Genomics of Infectious Diseases (ACEGID), Redeemer's University, Ede, Osun State, Nigeria **- Authors:** Oluniyi P.E., Ajogbasile F.V., Kayode A., Oguzie J., Olawoye I., Uwanibe J., Olumade T., Folarin O.A., Ihekweazu C., Happi C.T.
- **Accession ID:** EPI_ISL_487099 - **Originating Laboratory:** Nigeria Centre for Disease Control (NCDC) – **Submiting Laboratory:** African Centre of Excellence for Genomics of Infectious Diseases (ACEGID), Redeemer's University, Ede, Osun State, Nigeria **- Authors:** Oluniyi P.E., Ajogbasile F.V., Kayode A., Oguzie J., Olawoye I., Uwanibe J., Olumade T., Folarin O.A., Ihekweazu C., Happi C.T.
- **Accession ID:** EPI_ISL_487101 - **Originating Laboratory:** Nigeria Centre for Disease Control (NCDC) – **Submiting Laboratory:** African Centre of Excellence for Genomics of Infectious Diseases (ACEGID), Redeemer's University, Ede, Osun State, Nigeria **- Authors:** Oluniyi P.E., Ajogbasile F.V., Kayode A., Oguzie J., Olawoye I., Uwanibe J., Olumade T., Folarin O.A., Ihekweazu C., Happi C.T.
- **Accession ID:** EPI_ISL_487102 - **Originating Laboratory:** Nigeria Centre for Disease Control (NCDC) – **Submiting Laboratory:** African Centre of Excellence for Genomics of Infectious Diseases (ACEGID), Redeemer's University, Ede, Osun State, Nigeria **- Authors:** Oluniyi P.E., Ajogbasile F.V., Kayode A., Oguzie J., Olawoye I., Uwanibe J., Olumade T., Folarin O.A., Ihekweazu C., Happi C.T.
- **Accession ID:** EPI_ISL_487103 - **Originating Laboratory:** Nigeria Centre for Disease Control (NCDC) – **Submiting Laboratory:** African Centre of Excellence for Genomics of Infectious Diseases (ACEGID), Redeemer's University, Ede, Osun State, Nigeria **- Authors:** Oluniyi P.E., Ajogbasile F.V., Kayode A., Oguzie J., Olawoye I., Uwanibe J., Olumade T., Folarin O.A., Ihekweazu C., Happi C.T.
- **Accession ID:** EPI_ISL_487105 - **Originating Laboratory**: Nigeria Centre for Disease Control (NCDC) – **Submiting Laboratory:** African Centre of Excellence for Genomics of Infectious Diseases (ACEGID), Redeemer's University, Ede, Osun State, Nigeria **- Authors:** Oluniyi P.E., Ajogbasile F.V., Kayode A., Oguzie J., Olawoye I., Uwanibe J., Olumade T., Folarin O.A., Ihekweazu C., Happi C.T.
- **Accession ID:** EPI_ISL_487107 - **Originating Laboratory:** Nigeria Centre for Disease Control (NCDC) – **Submiting Laboratory:** African Centre of Excellence for Genomics of Infectious Diseases (ACEGID), Redeemer's University, Ede, Osun State, Nigeria **- Authors:** Oluniyi P.E., Ajogbasile F.V., Kayode A., Oguzie J., Olawoye I., Uwanibe J., Olumade T., Folarin O.A., Ihekweazu C., Happi C.T.
- **Accession ID:** EPI_ISL_487109 - **Originating Laboratory:** Nigeria Centre for Disease Control (NCDC) – **Submiting Laboratory:** African Centre of Excellence for Genomics of Infectious Diseases (ACEGID), Redeemer's University, Ede, Osun State, Nigeria **- Authors:** Oluniyi P.E., Ajogbasile F.V., Kayode A., Oguzie J., Olawoye I., Uwanibe J., Olumade T., Folarin O.A., Ihekweazu C., Happi C.T.
- **Accession ID:** EPI_ISL_417444 - **Originating Laboratory:** Department of Healthcare Biotechnology, National University of Sciences and Technology (NUST) – **Submiting Laboratory:** Department of Healthcare Biotechnology, National University of Sciences and Technology (NUST) **- Authors:** Javed, A., Niazi,S.K., Ghani,E., Saqib,M., Janjua,H.A., Corman,V.M. and Zohaib,A.
- **Accession ID:** EPI_ISL_419313 - **Originating Laboratory:** Molecular Biology and Biotechnology Lab II – **Submiting Laboratory:** Molecular Biology and Biotechnology Lab II **- Authors:** Tayyaba Zainab, Sana Shamshad, Azka Noureen, Aimen Malik, Muhammad Javaid Asad, Kumail Ali Rizvi
- **Accession ID:** EPI_ISL_451958 - **Originating Laboratory:** Jamil-ur-Rahman Center for Genome Research, Dr. Panjwani Center for Molecular Medicine and Drug Research, International Center for Chemical and Biological Sciences, University of Karachi – **Submiting Laboratory:** Jamil-ur-Rahman Center for Genome Research, Dr. Panjwani Center for Molecular Medicine and Drug Research, International Center for Chemical and Biological Sciences, University of Karachi **- Authors:** Shakeel,M., Raza,S.A., Khan,S., Khan,B.A., Zahid,M., Qureshi,M.A.and Khan,I.A
- **Accession ID:** EPI_ISL_468159 - **Originating Laboratory:** unknown – **Submiting Laboratory:** Department of Virology, Public Health Laboratories Division, National Institute of Health **- Authors:** Massab Umair, Aamer Ikram, Muhammad Salman, Adnan Khurshid, Nazish Badar, Shannon Whitmer, John Klena
- **Accession ID:** EPI_ISL_468162 - **Originating Laboratory:** unknown – **Submiting Laboratory:** Department of Virology, Public Health Laboratories Division, National Institute of Health **- Authors:** Massab Umair, Aamer Ikram, Muhammad Salman, Adnan Khurshid, Nazish Badar, Shannon Whitmer, John Klena
- **Accession ID:** EPI_ISL_468163 - **Originating Laboratory:** Department of Virology, Public Health Laboratories Division, National Institute of Health – **Submiting Laboratory:** Department of Virology, Public Health Laboratories Division, National Institute of Health **- Authors:** Massab Umair, Aamer Ikram, Muhammad Salman, Adnan Khurshid, Nazish Badar, Shannon Whitmer, John Klena
- **Accession ID:** EPI_ISL_476492 - **Originating Laboratory:** Institut Pasteur Dakar – **Submiting Laboratory:** Institut Pasteur de Dakar **- Authors:** Ndongo Dia, Moussa Moise Diagne, Mamadou Diop, Ousmane Faye, Amadou Alpha Sall
- **Accession ID:** EPI_ISL_476493 - **Originating Laboratory:** Institut Pasteur Dakar – **Submiting Laboratory:** Institut Pasteur de Dakar **- Authors:** Ndongo Dia, Moussa Moise Diagne, Mamadou Diop, Ousmane Faye, Amadou alpha Sall
- **Accession ID:** EPI_ISL_476516 - **Originating Laboratory**: Institut Pasteur Dakar – **Submiting Laboratory**: Institut Pasteur de Dakar **- Authors:** Ndongo Dia, Moussa Moise Diagne, mamadou Diop, Ousmane Faye, Amadou Alpha Sall
- **Accession ID:** EPI_ISL_476558 - **Originating Laboratory:** Institut Pasteur Dakar – **Submiting Laboratory:** Institut Pasteur de Dakar **- Authors:** Ndongo Dia, Moussa Moise Diagne, Mamadou Diop, Ousmane Faye, Amadou Alpha Sall
- **Accession ID:** EPI_ISL_476574 - **Originating Laboratory:** Institut Pasteur Dakar – **Submiting Laboratory:** Institut Pasteur de Dakar **- Authors:** Ndongo Dia, Moussa Moise Diagne, Mamadou Diop, Ousmane Faye, Amadou Alpha Sall
- **Accession ID:** EPI_ISL_480554 - **Originating Laboratory:** Institut Pasteur Dakar – **Submiting Laboratory:** Institut Pasteur de Dakar **- Authors:** Ndongo Dia, Moussa Moise Diagne, Mamadou Diop, Marie Henriette Dior Ndione, Mamadou Malado Jallow, Safietou Sanke, Ousmane Faye, Amadou Alpha Sall.
- **Accession ID:** EPI_ISL_480556 - **Originating Laboratory:** Institut Pasteur Dakar – **Submiting Laboratory:** Institut Pasteur de Dakar **- Authors:** Ndongo Dia, Moussa Moise Diagne, Mamadou Diop, Marie Henriette Dior Ndione, Mamadou Malado Jallow, Safietou Sanke, Ousmane Faye, Amadou Alpha Sall.
- **Accession ID:** EPI_ISL_480782 - **Originating Laboratory:** Institut Pasteur Dakar – **Submiting Laboratory**: Institut Pasteur de Dakar **- Authors:** Ndongo Dia, Moussa Moise Diagne, Mamadou Diop, Marie Henriette Dior Ndione, Mamadou Malado Jallow, Safietou Sanke, Ousmane Faye, Amadou Alpha Sall.
- **Accession ID:** EPI_ISL_481238 - **Originating Laboratory**: Institut Pasteur Dakar – **Submiting Laboratory**: Institut Pasteur de Dakar **- Authors:** Ndongo Dia, Moussa Moise Diagne, Mamadou Diop, Marie Henriette Dior Ndione, Mamadou Malado Jallow, Safietou Sanke, Ousmane Faye, Amadou Alpha Sall.
- **Accession ID:** EPI_ISL_482874 - **Originating Laboratory**: Institut Pasteur Dakar – **Submiting Laboratory**: Institut Pasteur de Dakar **- Authors:** Ndongo Dia, Moussa Moise Diagne, Mamadou Diop, Marie Henriette Dior Ndione, Mamadou malado Jallow, Safietou Sankhe, Ousmane Faye, Amadou Alpha Sall.
- **Accession ID:** EPI_ISL_482876 - **Originating Laboratory:** Institut Pasteur Dakar – **Submiting Laboratory:** Institut Pasteur de Dakar **- Authors:** Ndongo Dia, Moussa Moise Diagne, Mamadou Diop, Marie Henriette Dior Ndione, Mamadou malado Jallow, Safietou Sankhe, Ousmane Faye, Amadou Alpha Sall.
- **Accession ID:** EPI_ISL_482877 - **Originating Laboratory:** Institut Pasteur Dakar – **Submiting Laboratory**: Institut Pasteur de Dakar **- Authors:** Ndongo Dia, Moussa Moise Diagne, Mamadou Diop, Marie Henriette Dior Ndione, Mamadou malado Jallow, Safietou Sankhe, Ousmane Faye, Amadou Alpha Sall.
- **Accession ID:** EPI_ISL_512812 - **Originating Laboratory**: Kenema Government Hospital, Ministry of Health and Sanitation – **Submiting Laboratory:** Kenema Government Hospital, Ministry of Health and Sanitation **- Authors:** Goba,A., Momoh,M., Sandi,J., Tomkins-Tinch,C., Siddle,K., Mehta,S., Oluniyi,P., Jalloh,S., Park,D., Andersen,K., Garry,R., Happi,C., Grant,D., Olawoye,I.
- **Accession ID:** EPI_ISL_512813 - **Originating Laboratory:** Kenema Government Hospital, Ministry of Health and Sanitation – **Submiting Laboratory:** Kenema Government Hospital, Ministry of Health and Sanitation **- Authors:** Goba,A., Momoh,M., Sandi,J., Tomkins-Tinch,C., Siddle,K., Mehta,S., Oluniyi,P., Jalloh,S., Park,D., Andersen,K., Garry,R., Happi,C., Grant,D., Olawoye,I.
- **Accession ID:** EPI_ISL_512814 - **Originating Laboratory:** Kenema Government Hospital, Ministry of Health and Sanitation – **Submiting Laboratory:** Kenema Government Hospital, Ministry of Health and Sanitation **- Authors:** Goba,A., Momoh,M., Sandi,J., Tomkins-Tinch,C., Siddle,K., Mehta,S., Oluniyi,P., Jalloh,S., Park,D., Andersen,K., Garry,R., Happi,C., Grant,D., Olawoye,I.
- **Accession ID:** EPI_ISL_512816 - **Originating Laboratory:** Kenema Government Hospital, Ministry of Health and Sanitation – **Submiting Laboratory:** Kenema Government Hospital, Ministry of Health and Sanitation **- Authors:** Goba,A., Momoh,M., Sandi,J., Tomkins-Tinch,C., Siddle,K., Mehta,S., Oluniyi,P., Jalloh,S., Park,D., Andersen,K., Garry,R., Happi,C., Grant,D., Olawoye,I.
- **Accession ID:** EPI_ISL_512817 - **Originating Laboratory:** Kenema Government Hospital, Ministry of Health and Sanitation – **Submiting Laboratory:** Kenema Government Hospital, Ministry of Health and Sanitation **- Authors:** Goba,A., Momoh,M., Sandi,J., Tomkins-Tinch,C., Siddle,K., Mehta,S., Oluniyi,P., Jalloh,S., Park,D., Andersen,K., Garry,R., Happi,C., Grant,D., Olawoye,I.
- **Accession ID:** EPI_ISL_512818 - **Originating Laboratory:** Kenema Government Hospital, Ministry of Health and Sanitation – **Submiting Laboratory:** Kenema Government Hospital, Ministry of Health and Sanitation **- Authors:** Goba,A., Momoh,M., Sandi,J., Tomkins-Tinch,C., Siddle,K., Mehta,S., Oluniyi,P., Jalloh,S., Park,D., Andersen,K., Garry,R., Happi,C., Grant,D., Olawoye,I.
- **Accession ID:** EPI_ISL_512819 - **Originating Laboratory:** Kenema Government Hospital, Ministry of Health and Sanitation – **Submiting Laboratory:** Kenema Government Hospital, Ministry of Health and Sanitation **- Authors:** Goba,A., Momoh,M., Sandi,J., Tomkins-Tinch,C., Siddle,K., Mehta,S., Oluniyi,P., Jalloh,S., Park,D., Andersen,K., Garry,R., Happi,C., Grant,D., Olawoye,I.
- **Accession ID:** EPI_ISL_512820 - **Originating Laboratory:** Kenema Government Hospital, Ministry of Health and Sanitation – **Submiting Laboratory**: Kenema Government Hospital, Ministry of Health and Sanitation **- Authors:** Goba,A., Momoh,M., Sandi,J., Tomkins-Tinch,C., Siddle,K., Mehta,S., Oluniyi,P., Jalloh,S., Park,D., Andersen,K., Garry,R., Happi,C., Grant,D., Olawoye,I.
- **Accession ID:** EPI_ISL_512821 - **Originating Laboratory:** Kenema Government Hospital, Ministry of Health and Sanitation – **Submiting Laboratory:** Kenema Government Hospital, Ministry of Health and Sanitation **- Authors:** Goba,A., Momoh,M., Sandi,J., Tomkins-Tinch,C., Siddle,K., Mehta,S., Oluniyi,P., Jalloh,S., Park,D., Andersen,K., Garry,R., Happi,C., Grant,D., Olawoye,I.
- **Accession ID:** EPI_ISL_430297 - **Originating Laboratory:** National Institute for Communicable Diseases of the National Health Laboratory Service – **Submiting Laboratory:** National Institute for Communicable Diseases of the National Health Laboratory Service **- Authors:** Allam M, Kwenda S, van Heusden P, Khumalo Z, Mohale T, Subramoney K, von Gottberg, A, Ismail A, Bhiman JN
- **Accession ID:** EPI_ISL_455633 - **Originating Laboratory:** KRISP, KZN Research Innovation and Sequencing Platform – **Submiting Laboratory:** KRISP, KZN Research Innovation and Sequencing Platform **- Authors:** Giandhari J, Pillay S, Lessells R, Chimukangara B, Deforche K, Tegally H, Wilkinson E, de Oliveira T
- **Accession ID:** EPI_ISL_467461 - **Originating Laboratory:** AMPATH-DBN – **Submiting Laboratory:** KRISP, KZN Research Innovation and Sequencing Platform **- Authors:** Giandhari J, Pillay S, Lessells R, Chimukangara B, Mdlalose K, York D, Khan S, Tegally H, Wilkinson E, de Oliveira T
- **Accession ID:** EPI_ISL_487337 - **Originating Laboratory:** Molecular Diagnostics Services (MDS) – **Submiting Laboratory:** KRISP, KZN Research Innovation and Sequencing Platform **- Authors:** Giandhari J, Pillay S, Lessells R, Chimukangara B, Mdlalose K, York D, Khan S, Tegally H, Wilkinson E, de Oliveira T
- **Accession ID:** EPI_ISL_498055 - **Originating Laboratory:** NHLS-IALCH – **Submiting Laboratory:** KRISP, KZN Research Innovation and Sequencing Platform **- Authors:** Giandhari J, Pillay S, Lessells R, Chimukangara B, Mdlalose K, York D, Khan S, Tegally H, Wilkinson E, de Oliveira T
- **Accession ID:** EPI_ISL_515844 - **Originating Laboratory:** Medical Disagnostics Services (MDS) – **Submiting Laboratory:** KRISP, KZN Research Innovation and Sequencing Platform **- Authors:** Giandhari J, Pillay S, Lessells R, ChimukangaraB, Mdlalose K, York D, Khan S, Tegally H, Wilkinson E, de Oliveira T
- **Accession ID:** EPI_ISL_515847 - **Originating Laboratory:** Medical Disagnostics Services (MDS) – **Submiting Laboratory:** KRISP, KZN Research Innovation and Sequencing Platform **- Authors:** Giandhari J, Pillay S, Lessells R, ChimukangaraB, Mdlalose K, York D, Khan S, Tegally H, Wilkinson E, de Oliveira T
- **Accession ID:** EPI_ISL_535417 - **Originating Laboratory:** NHLS-IALCH – **Submiting Laboratory:** KRISP, KZN Research Innovation and Sequencing Platform **- Authors:** Giandhari J, Pillay S, Lessells R, Mdlalose K, York D, Khan S, Tegally H, Wilkinson E, de Oliveira T
- **Accession ID:** EPI_ISL_535435 - **Originating Laboratory:** NHLS-IALCH – **Submiting Laboratory:** KRISP, KZN Research Innovation and Sequencing Platform **- Authors:** Giandhari J, Pillay S, Lessells R, Mdlalose K, York D, Khan S, Tegally H, Wilkinson E, de Oliveira T
- **Accession ID:** EPI_ISL_535457 - **Originating Laboratory:** NHLS-IALCH – **Submiting Laboratory:** KRISP, KZN Research Innovation and Sequencing Platform **- Authors:** Giandhari J, Pillay S, Lessells R, Mdlalose K, York D, Khan S, Tegally H, Wilkinson E, de Oliveira T
- **Accession ID:** EPI_ISL_535496 - **Originating Laboratory:** NHLS-IALCH – **Submiting Laboratory:** KRISP, KZN Research Innovation and Sequencing Platform **- Authors:** Giandhari J, Pillay S, Lessells R, Mdlalose K, York D, Khan S, Tegally H, Wilkinson E, de Oliveira T
- **Accession ID:** EPI_ISL_535511 - **Originating Laboratory:** NHLS-IALCH – **Submiting Laboratory:** KRISP, KZN Research Innovation and Sequencing Platform **- Authors:** Giandhari J, Pillay S, Lessells R, Mdlalose K, York D, Khan S, Tegally H, Wilkinson E, de Oliveira T
- **Accession ID:** EPI_ISL_535533 - **Originating Laboratory:** NHLS-IALCH – **Submiting Laboratory:** KRISP, KZN Research Innovation and Sequencing Platform **- Authors:** Giandhari J, Pillay S, Lessells R, Mdlalose K, York D, Khan S, Tegally H, Wilkinson E, de Oliveira T
- **Accession ID:** EPI_ISL_535539 - **Originating Laboratory:** NHLS-IALCH – **Submiting Laboratory:** KRISP, KZN Research Innovation and Sequencing Platform **- Authors:** Giandhari J, Pillay S, Lessells R, Mdlalose K, York D, Khan S, Tegally H, Wilkinson E, de Oliveira T
- **Accession ID:** EPI_ISL_535544 - **Originating Laboratory:** NHLS-IALCH – **Submiting Laboratory:** KRISP, KZN Research Innovation and Sequencing Platform **- Authors:** Giandhari J, Pillay S, Lessells R, Mdlalose K, York D, Khan S, Tegally H, Wilkinson E, de Oliveira T
- **Accession ID:** EPI_ISL_535558 - **Originating Laboratory:** NHLS-IALCH – **Submiting Laboratory:** KRISP, KZN Research Innovation and Sequencing Platform **- Authors:** Giandhari J, Pillay S, Lessells R, Mdlalose K, York D, Khan S, Tegally H, Wilkinson E, de Oliveira T
- **Accession ID:** EPI_ISL_535569 - **Originating Laboratory:** NHLS-IALCH – **Submiting Laboratory:** KRISP, KZN Research Innovation and Sequencing Platform **- Authors:** Giandhari J, Pillay S, Lessells R, Mdlalose K, York D, Khan S, Tegally H, Wilkinson E, de Oliveira T
- **Accession ID:** EPI_ISL_458286 - **Originating Laboratory:** unknown – **Submiting Laboratory:** Bundeswehr Institute of Microbiology **- Authors:** Handrick,S., Bestehorn-Willmann,M.S., Eckstein,S., Walter,M.C., Antwerpen,M.H., Rehn,A., Naija,H., Stoecker,K., Woelfel,R. and Ben Moussa,M.
- **Accession ID:** EPI_ISL_463001 - **Originating Laboratory:** unknown – **Submiting Laboratory:** Clinical virology, Institut Pasteur de Tunis **- Authors:** Fares, W., Triki, H.
- **Accession ID:** EPI_ISL_463002 - **Originating Laboratory:** unknown – **Submiting Laboratory:** Clinical virology, Institut Pasteur de Tunis **- Authors:** Fares, W., Triki, H.
- **Accession ID:** EPI_ISL_463003 - **Originating Laboratory:** unknown – **Submiting Laboratory:** Clinical virology, Institut Pasteur de Tunis **- Authors:** Fares, W., Triki, H.
- **Accession ID:** EPI_ISL_463005 - **Originating Laboratory:** unknown – **Submiting Laboratory:** Clinical virology, Institut Pasteur de Tunis **- Authors:** Fares, W., Triki, H.
- **Accession ID:** EPI_ISL_463006 - **Originating Laboratory:** unknown – **Submiting Laboratory:** Clinical virology, Institut Pasteur de Tunis **- Authors:** Fares, W., Triki, H.
- **Accession ID:** EPI_ISL_451183 - **Originating Laboratory:** Uganda Virus Research Institute – **Submiting Laboratory:** MRC/UVRI & LSHTM Uganda Research Unit **- Authors:** Dan Lule Bugembe, John Kayiwa, My V.T Phan, Phionah Tushabe, Stephen Balinandi, Beatrice Dhaala, Deogratius Ssemwanga, Jonas Lexow, Henry Mwebesa, Jane Aceng, Henry Kyobe, Julius Lutwama, Pontiano Kaleebu, Matthew Cotten
- **Accession ID:** EPI_ISL_451186 - **Originating Laboratory:** Uganda Virus Research Institute – **Submiting Laboratory:** MRC/UVRI & LSHTM Uganda Research Unit **- Authors:** Dan Lule Bugembe, John Kayiwa, My V.T Phan, Phionah Tushabe, Stephen Balinandi, Beatrice Dhaala, Deogratius Ssemwanga, Jonas Lexow, Henry Mwebesa, Jane Aceng, Henry Kyobe, Julius Lutwama, Pontiano Kaleebu, Matthew Cotten
- **Accession ID:** EPI_ISL_451189 - **Originating Laboratory:** Uganda Virus Research Institute – **Submiting Laboratory:** MRC/UVRI & LSHTM Uganda Research Unit **- Authors:** Dan Lule Bugembe, John Kayiwa, My V.T Phan, Phionah Tushabe, Stephen Balinandi, Beatrice Dhaala, Deogratius Ssemwanga, Jonas Lexow, Henry Mwebesa, Jane Aceng, Henry Kyobe, Julius Lutwama, Pontiano Kaleebu, Matthew Cotten
- **Accession ID:** EPI_ISL_451194 - **Originating Laboratory:** Uganda Virus Research Institute – **Submiting Laboratory:** MRC/UVRI & LSHTM Uganda Research Unit **- Authors:** Dan Lule Bugembe, John Kayiwa, My V.T Phan, Phionah Tushabe, Stephen Balinandi, Beatrice Dhaala, Deogratius Ssemwanga, Jonas Lexow, Henry Mwebesa, Jane Aceng, Henry Kyobe, Julius Lutwama, Pontiano Kaleebu, Matthew Cotten
- **Accession ID:** EPI_ISL_451195 - **Originating Laboratory:** Uganda Virus Research Institute – **Submiting Laboratory:** MRC/UVRI & LSHTM Uganda Research Unit **- Authors:** Dan Lule Bugembe, John Kayiwa, My V.T Phan, Phionah Tushabe, Stephen Balinandi, Beatrice Dhaala, Deogratius Ssemwanga, Jonas Lexow, Henry Mwebesa, Jane Aceng, Henry Kyobe, Julius Lutwama, Pontiano Kaleebu, Matthew Cotten
- **Accession ID:** EPI_ISL_451202 - **Originating Laboratory:** Uganda Virus Research Institute – **Submiting Laboratory:** MRC/UVRI & LSHTM Uganda Research Unit **- Authors:** Dan Lule Bugembe, John Kayiwa, My V.T Phan, Phionah Tushabe, Stephen Balinandi, Beatrice Dhaala, Deogratius Ssemwanga, Jonas Lexow, Henry Mwebesa, Jane Aceng, Henry Kyobe, Julius Lutwama, Pontiano Kaleebu, Matthew Cotten
- **Accession ID:** EPI_ISL_510529 - **Originating Laboratory:** School of Veterinary Medicine, Disease Control – **Submiting Laboratory:** School of Veterinary Medicine, Disease Control **- Authors:** Simulundu,E., Kapata,N., Mupeta,F., Kapata,P.C., Saasa,N., Changula,K., Muleya,W., Chitanga,S., Chambaro,H., Mubemba,B., Masahiro,K., Chanda,D., Mulenga,L., Fwoloshi,S., Shibemba,A.L., Kapaya,F., Zulu,P., Musonda,K., Monze,M., Sinyange,N., Liwewe,M.M., Kapin'a,M., Chipimo,P.J., Ngosa,W., Morales,A.N., Kayeyi,N., Malama, K., Tembo,J., Bates,M., Sawa, H., Takada,A., Nalubamba,K.S., Mukonka,V., Chilufya,C. and Zumla, A.
- **Accession ID:** EPI_ISL_487276 - **Originating Laboratory:** Department of Food Safety, Nutrition and Veterinary public health, Istituto Superiore di Sanita' – **Submiting Laboratory:** Department of Biomedical, Surgical and Dental Sciences and Department of Biomedical Sciences for Health **- Authors:** Delbue,S., Ferrante,P., Basilico,N., Parapini,S., Binda,S., D'Alessandro,S., Galli,C., Signorini,L., Primache,V., Anselmi,G., Pariani,E.
- **Accession ID:** EPI_ISL_492981 - **Originating Laboratory**: IRCCS Sacro Cuore Don Calabria Hospital, Department of Infectious, Tropical Diseases & Microbiology – **Submiting Laboratory:** University of Verona, Department of Biotechnology **- Authors:** Antonio Mori, Michela Deiana, Elena Pomari, Chiara Piubelli; Giulia Lopatriello, Luca Marcolungo, Cristina Beltrami, Chiara Degli Esposti, Emanuela Cosentino, Massimo Delledonne
- **Accession ID:** EPI_ISL_493330 - **Originating Laboratory:** INMI Lazzaro Spallanzani IRCCS – **Submiting Laboratory:** INMI Lazzaro Spallanzani IRCCS **- Authors:** Cesare E.M. Gruber, Martina Rueca, Barbara Bartolini, Francesco Messina, Maria R. Capobianchi, Antonino Di Caro
- **Accession ID:** EPI_ISL_493332 - **Originating Laboratory**: Istituto Zooprofilattico Sperimentale del Mezzogiorno – **Submiting Laboratory:** INMI Lazzaro Spallanzani IRCCS **- Authors:** Cesare E.M. Gruber, Martina Rueca, Barbara Bartolini, Francesco Messina, Antonino Di Caro, Giovanna Fusco, Maurizio Viscardi, Giorgia Borriello, Maria R. Capobianchi
- **Accession ID:** EPI_ISL_494757 - **Originating Laboratory**: INT Fondazione Pascale – **Submiting Laboratory:** INT Fondazione Pascale **- Authors:** INT Fondazione Pascale
- **Accession ID:** EPI_ISL_516085 - **Originating Laboratory:** Biomedical Sciences and Public Health, Polytechnic University of Marche – **Submiting Laboratory:** Biomedical Sciences and Public Health, Polytechnic University of Marche **- Authors:** Bagnarelli, P., Caucci,S., Di Sante,L., Menzo,S., Alessandrini,F., Onofri,V., Turchi,C., Melchionda,F., Tagliabracci,A.
- **Accession ID:** EPI_ISL_522863 - **Originating Laboratory:** ULSS9 Distretto di Bussolengo – **Submiting Laboratory:** Istituto Zooprofilattico Sperimentale delle Venezie **- Authors:** Adelaide Milani, Alessia Schivo, Annalisa Salviato, Erika Giorgia Quaranta, Gianpiero Zamperin, Ambra Pastori, Bianca Zecchin, Alice Fusaro, Calogero Terregino, Antonia Ricci
- **Accession ID:** EPI_ISL_525495 - **Originating Laboratory**: Laboratory of Molecular Virology of the International Centre for Genetic Engineering and Biotechnology (ICGEB) – **Submiting Laboratory:** ARGO Open Lab Platform for Genome Sequencing **- Authors:** Licastro D, Rajasekharan S, Dal Monego S, Segat L, D'Agaro P, Marcello A
- **Accession ID:** EPI_ISL_525553 - **Originating Laboratory:** Istituto Zooprofilattico Sperimentale Puglia e Basilicata; Dipartimento di Bioscienze, Biotecnologie e Biofarmaceutica dell’Università degli Studi di Bari “A. Moro”; Istituto di Biomembrane. Bioenergetica e Biotecnologie Molecolari del Consiglio Nazionale delle Ricerche di Bari – **Submiting Laboratory:** Beaconlab (Bioinformatics, Evolution and Comparative Genomics lab), Dept of Biosciences, University on Milan **- Authors:** Parisi A.,Pesole G., Manzari C., Chiara M
- **Accession ID:** EPI_ISL_525555 - **Originating Laboratory:** Istituto Zooprofilattico Sperimentale Puglia e Basilicata; Dipartimento di Bioscienze, Biotecnologie e Biofarmaceutica dell’Università degli Studi di Bari “A. Moro”; Istituto di Biomembrane. Bioenergetica e Biotecnologie Molecolari del Consiglio Nazionale delle Ricerche di Bari – **Submiting Laboratory:** Beaconlab (Bioinformatics, Evolution and Comparative Genomics lab), Dept of Biosciences, University on Milan  **- Authors:** Parisi A.,Pesole G., Manzari C., Chiara M
- **Accession ID:** EPI_ISL_528925 - **Originating Laboratory:** Ospedale Regionale San Salvatore-L'Aquila – **Submiting Laboratory:** Istitituto Zooprofilattico Sperimentale dell'Abruzzo e Molise "G. Caporale" **- Authors:** Lorusso A, Marcacci M, Di Domenico M, Curini V, Ancora M, Cammà C, Rinaldi A, Mangone I, Di Pasquale A, Puglia I, Savini G.
- **Accession ID:** EPI_ISL_529026 - **Originating Laboratory:** Ospedale "Giuseppe Mazzini"-Teramo – **Submiting Laboratory:** Istitituto Zooprofilattico Sperimentale dell'Abruzzo e Molise "G. Caporale" **- Authors:** Lorusso A, Marcacci M, Di Domenico M, Curini V, Ancora M, Cammà C, Rinaldi A, Mangone I, Di Pasquale A, Puglia I, Savini G.
- **Accession ID:** EPI_ISL_412974 - **Originating Laboratory:** Department of Infectious Diseases, Istituto Superiore di Sanità, Rome, Italy – **Submiting Laboratory:** Virology Laboratory, Scientific Department, Army Medical Center **- Authors:** Paola Stefanelli, Stefano Fiore, Antonella Marchi, Eleonora Benedetti, Concetta Fabiani, Giovanni Faggioni, Antonella Fortunato, Silvia Fillo, Riccardo De Santis, Andrea Ciammaruconi, Giancarlo Petralito, Filippo Molinari, Florigio Lista
- **Accession ID:** EPI_ISL_412973 - **Originating Laboratory:** Department of Infectious Diseases, Istituto Superiore di Sanità, Roma, Italy – **Submiting Laboratory:** Virology Laboratory, Scientific Department, Army Medical Center **- Authors:** Paola Stefanelli, Stefano Fiore, Antonella Marchi, Eleonora Benedetti, Concetta Fabiani, Giovanni Faggioni, Antonella Fortunato, Riccardo De Santis, Silvia Fillo, Anna Anselmo, Andrea Ciammaruconi, Stefano Palomba, Florigio Lista
- **Accession ID:** EPI_ISL_457699 - **Originating Laboratory:** Department of Infectious Diseases, Istituto Superiore di Sanità, Roma, Italy – **Submiting Laboratory:** Army Medical and Veterinary Research Center **- Authors:** Paola Stefanelli, Alessandra Lo Presti, Stefano Fiore, Antonella Marchi, Eleonora Benedetti, Concetta Fabiani Silvia Fillo, Giovanni Faggioni, Riccardo De Sanctis, Antonella Fortunato, Anna Anselmo, Francesco Giordani, Vanessa Vera Fain, Nino D’Amore, Florigio Lista
- **Accession ID:** EPI_ISL_457700 - **Originating Laboratory:** Department of Infectious Diseases, Istituto Superiore di Sanità, Roma, Italy – **Submiting Laboratory:** Army Medical and Veterinary Research Center **- Authors:** Paola Stefanelli, Alessandra Lo Presti, Stefano Fiore, Antonella Marchi, Eleonora Benedetti, Concetta Fabiani Silvia Fillo, Giovanni Faggioni, Riccardo De Sanctis, Antonella Fortunato, Anna Anselmo, Francesco Giordani, Vanessa Vera Fain, Nino D’Amore, Florigio Lista
- **Accession ID:** EPI_ISL_457724 - **Originating Laboratory:** Department of Infectious Diseases, Istituto Superiore di Sanità, Roma, Italy – **Submiting Laboratory:** Army Medical and Veterinary Research Center **- Authors:** Paola Stefanelli, Alessandra Lo Presti, Stefano Fiore, Antonella Marchi, Eleonora Benedetti, Concetta Fabiani Silvia Fillo, Giovanni Faggioni, Riccardo De Sanctis, Antonella Fortunato, Anna Anselmo, Francesco Giordani, Vanessa Vera Fain, Nino D’Amore, Florigio Lista
- **Accession ID:** EPI_ISL_457728 - **Originating Laboratory**: Department of Infectious Diseases, Istituto Superiore di Sanità, Roma, Italy – **Submiting Laboratory:** Army Medical and Veterinary Research Center **- Authors:** Paola Stefanelli, Alessandra Lo Presti, Stefano Fiore, Antonella Marchi, Eleonora Benedetti, Concetta Fabiani Silvia Fillo, Giovanni Faggioni, Riccardo De Sanctis, Antonella Fortunato, Anna Anselmo, Francesco Giordani, Vanessa Vera Fain, Nino D’Amore, Florigio Lista
- **Accession ID:** EPI_ISL_457732 - **Originating Laboratory:** Department of Infectious Diseases, Istituto Superiore di Sanità, Roma, Italy – **Submiting Laboratory:** Army Medical and Veterinary Research Center **- Authors:** Paola Stefanelli, Alessandra Lo Presti, Stefano Fiore, Antonella Marchi, Eleonora Benedetti, Concetta Fabiani Silvia Fillo, Giovanni Faggioni, Riccardo De Sanctis, Antonella Fortunato, Anna Anselmo, Francesco Giordani, Vanessa Vera Fain, Nino D’Amore, Florigio Lista
- **Accession ID:** EPI_ISL_457736 - **Originating Laboratory:** Department of Infectious Diseases, Istituto Superiore di Sanità, Roma, Italy – **Submiting Laboratory:** Army Medical and Veterinary Research Center **- Authors:** Paola Stefanelli, Alessandra Lo Presti, Stefano Fiore, Antonella Marchi, Eleonora Benedetti, Concetta Fabiani Silvia Fillo, Giovanni Faggioni, Riccardo De Sanctis, Antonella Fortunato, Anna Anselmo, Francesco Giordani, Vanessa Vera Fain, Nino D’Amore, Florigio Lista
- **Accession ID:** EPI_ISL_457749 - **Originating Laboratory:** Department of Infectious Diseases, Istituto Superiore di Sanità, Roma, Italy – **Submiting Laboratory:** Army Medical and Veterinary Research Center **- Authors:** Paola Stefanelli, Alessandra Lo Presti, Stefano Fiore, Antonella Marchi, Eleonora Benedetti, Concetta Fabiani Silvia Fillo, Giovanni Faggioni, Riccardo De Sanctis, Antonella Fortunato, Anna Anselmo, Francesco Giordani, Vanessa Vera Fain, Nino D’Amore, Florigio Lista
- **Accession ID:** EPI_ISL_ 486858 - **Originating Laboratory:** Institut Pasteur Dakar - Senegal – **Submiting Laboratory:** Institut Pasteur de Dakar– Senegal **Authors:** Ndongo Dia, Moussa Moise Diagne, Mamadou Diop, Marie Henriette Dior Ndione, Mamadou Malado Jallow, Safietou Sanke, Ousmane Faye, Amadou Alpha Sall.
- **Accession ID:** EPI_ISL_ 486864 - **Originating Laboratory:** Institut Pasteur Dakar - Senegal – **Submiting Laboratory:** Institut Pasteur Dakar - Senegal **Authors:** Ndongo Dia, Moussa Moise Diagne, Mamadou Diop, Marie Henriette Dior Ndione, Mamadou Malado Jallow, Safietou Sanke, Ousmane Faye, Amadou Alpha Sall.
- **Accession ID:** EPI_ISL_ 486865 - **Originating Laboratory:** Institut Pasteur Dakar - Senegal – **Submiting Laboratory:** Institut Pasteur Dakar - Senegal **Authors:** Ndongo Dia, Moussa Moise Diagne, Mamadou Diop, Marie Henriette Dior Ndione, Mamadou Malado Jallow, Safietou Sanke, Ousmane Faye, Amadou Alpha Sall.
- **Accession ID:** EPI_ISL_ 498232 - **Originating Laboratory:** Institut Pasteur Dakar - Senegal – **Submiting Laboratory:** Institut Pasteur Dakar - Senegal **Authors:** Ndongo Dia, Moussa Moise Diagne, Mamadou Diop, Marie Henriette Dior Ndione, Mamadou Malado Jallow, Safietou Sanke, Ousmane Faye, Amadou Alpha Sall.
- **Accession ID:** EPI_ISL_ 498234 - **Originating Laboratory:** Institut Pasteur Dakar - Senegal – **Submiting Laboratory:** Institut Pasteur Dakar - Senegal **Authors:** Ndongo Dia, Moussa Moise Diagne, Mamadou Diop, Marie Henriette Dior Ndione, Mamadou Malado Jallow, Safietou Sankhe Mbengue, Ousmane Faye, Amadou Alpha Sall.
- **Accession ID:** EPI_ISL_498238 - **Originating Laboratory:** Institut Pasteur Dakar - Senegal – **Submiting Laboratory:** Institut Pasteur Dakar - Senegal **Authors:** dongo Dia, Moussa Moise Diagne, Mamadou Diop, Marie Henriette Dior Ndione, Mamadou Malado Jallow, Safietou Sankhe Mbengue, Ousmane Faye, Amadou Alpha Sall.
- **Accession ID:** EPI_ISL_498239 - **Originating Laboratory:** Institut Pasteur Dakar - Senegal – **Submiting Laboratory:** Institut Pasteur Dakar - Senegal **Authors:** Ndongo Dia, Moussa Moise Diagne, Mamadou Diop, Marie Henriette Dior Ndione, Mamadou Malado Jallow, Safietou Sankhe Mbengue, Ousmane Faye, Amadou Alpha Sall.
- **Accession ID:** EPI_ISL_498252 - **Originating Laboratory:** Institut Pasteur Dakar - Senegal – **Submiting Laboratory:** Institut Pasteur Dakar - Senegal **Authors:** Ndongo Dia, Moussa Moise Diagne, Mamadou Diop, Marie Henriette Dior Ndione, Mamadou Malado Jallow, Safietou Sankhe Mbengue, Ousmane Faye, Amadou Alpha Sall.
- **Accession ID:** EPI_ISL_512811 - **Originating Laboratory:** Kenema Government Hospital, Ministry of Health and Sanitation, Kenema, Sierra Leone – **Submiting Laboratory:** Kenema Government Hospital, Ministry of Health and Sanitation, Kenema, Sierra Leone **Authors:** Goba,A., Momoh,M., Sandi,J., Tomkins-Tinch,C., Siddle,K., Mehta,S., Oluniyi,P., Jalloh,S., Park,D., Andersen,K., Garry,R., Happi,C., Grant,D., Olawoye,I.

*We gratefully acknowledge the Authors, the Originating and Submitting Laboratories for their sequence and metadata shared through NCBI (https://www.ncbi.nlm.nih.gov/nuccore), on which this research is based.*

- **Accession** **Number:** NC0445512 - Wuhan seafood market pneumonia virus isolate Wuhan-Hu-1, complete genome. Dec-2019. Shanghai Public Health Clinical Center & School of Public Health, Fudan University, Shanghai, China. Wu,F., Zhao,S., Yu,B., Chen,Y.-M., Wang,W., Hu,Y., Song,Z.-G., Tao,Z.-W., Tian,J.-H., Pei,Y.-Y., Yuan,M.L., Zhang,Y.-L., Dai,F.-H., Liu,Y., Wang,Q.-M., Zheng,J.-J., Xu,L., Holmes,E.C. and Zhang,Y.-Z.
- **Accession Number:** MT955171 - Severe acute respiratory syndrome coronavirus 2 isolate SARS-CoV-/human/TUN/TUN_ADAGE_4870/2020, complete genome. Molecular and Cellular Screening Process Laboratory, Center of Biotechnology of Sfax, Sidi Mansour, Sfax, Tunisia. Souissi,A., Abid,N., Ben Ayed,I., Gargouri,S., Abdelmoulah,F., Elargoubi,A., Smeti,I., Bensaid,M., Stambouli,N., Kharat,N., Ajili,F., Fki-berrajah,L., Mhalla,S., Chtourou,A., Gaaloul,I., Nabli,A., Turki,M., Aouni,M., Hammami,A., Mastouri,M., Karray Hakim,H., Kamoun,S., Rebai,A. and Masmoudi,S.
- **Accession Number**: MT502774 - Severe acute respiratory syndrome coronavirus 2 isolate SARS-CoV-2/Human/BGD/BARJ_CVASU_CTG_503/2020, complete genome. Bangladesh Jute Research Institute, Manik Mia Avenue, Dhaka, Bangladesh. Ahmed,R., Hossain,M.S., Kabir,S.M.T., Emdad,E.M., Haq,M.N.,Ahmed,E., Biswas,P.K., Chowdhury,M.A.H., Ahmed,M.S., Rahman,M.S., Huq, A.S.M.A., Das,G.B., Siddiki,A.Z., Haque,M.S., Alam,M.M. and Islam,M.S
- **Accession Number**: MT872492 - Severe acute respiratory syndrome coronavirus 2 isolate SARS-CoV-2/human/SLE/KGH-G-8626/2020, complete genome. Kenema Government Hospital, Sierra Leone. Goba, A., Momoh,M., Sandi,J., Tomkins-Tinch,C., Siddle,K., Mehta,S., Oluniyi,P., Olawoye,I., Jalloh,S., Park,D., Sabeti,P., Andersen,K., Garry,R., Happi,C. and Grant,D.
